# Biofilm microenvironment induces a widespread adaptive amino-acid fermentation pathway conferring strong fitness advantage in *Escherichia coli*

**DOI:** 10.1371/journal.pgen.1006800

**Published:** 2017-05-19

**Authors:** Sylvie Létoffé, Sabina Chalabaev, José Dugay, Franziska Stressmann, Bianca Audrain, Jean-Charles Portais, Fabien Letisse, Jean-Marc Ghigo

**Affiliations:** 1 Institut Pasteur, Genetics of Biofilms Laboratory. 25–28 rue du Docteur Roux, France; 2 Analytical, Bioanalytical Sciences and Miniaturization Laboratory, CNRS UMR CBI 8231, ESPCI Paris, 10 rue Vauquelin, Paris, France; 3 LISBP, Université de Toulouse, CNRS, INRA, INSA, Toulouse, France; Uppsala University, SWEDEN

## Abstract

Bacterial metabolism has been studied primarily in liquid cultures, and exploration of other natural growth conditions may reveal new aspects of bacterial biology. Here, we investigate metabolic changes occurring when *Escherichia coli* grows as surface-attached biofilms, a common but still poorly characterized bacterial lifestyle. We show that *E*. *coli* adapts to hypoxic conditions prevailing within biofilms by reducing the amino acid threonine into 1-propanol, an important industrial commodity not known to be naturally produced by *Enterobacteriaceae*. We demonstrate that threonine degradation corresponds to a fermentation process maintaining cellular redox balance, which confers a strong fitness advantage during anaerobic and biofilm growth but not in aerobic conditions. Whereas our study identifies a fermentation pathway known in *Clostridia* but previously undocumented in *Enterobacteriaceae*, it also provides novel insight into how growth in anaerobic biofilm microenvironments can trigger adaptive metabolic pathways edging out competition with in mixed bacterial communities.

## Author summary

Whereas *Escherichia coli* does not naturally produce the 1-propanol unless subjected to extensive genetic modifications, we show that this important industrial commodity is produced in hypoxic conditions inside biofilms. 1-propanol production corresponds to a native threonine fermentation pathway previously undocumented in *E*. *coli* and other *Enterobacteriaceae*. This widespread adaptive response contributes to maintain cellular redox balance and bacterial fitness in biofilms and other amino acid-rich hypoxic environments. This study therefore shows that mining complex lifestyles such as biofilm microenvironments provides new insight into the extent of bacterial metabolic potential and adaptive bacterial physiological responses.

## Introduction

Bacteria rapidly adapt to changes in available resources and environmental fluctuations due to their remarkable ability to finely tune their physiology and metabolism [1, 2]. Although bacterial adaptations have been primarily studied in liquid cultures composed of free-swimming planktonic cells, exploration of a broader range of growth conditions can reveal new aspects of bacterial biology [3]. One of the most common bacterial lifestyles corresponds to surface-attached communities called biofilms, in which high cell density, reduced diffusion and physico-chemical heterogeneity are associated with extensive physiological changes [4, 5]. Biofilm bacteria display different properties compared to planktonic bacteria, and biofilms have long been considered potential reservoirs of unknown functions, contributing to their ability to thrive on surfaces present in natural and anthropic environments [6]. It was, for example, hypothesized early on that the study of physiological adjustments occurring during biofilm formation would provide insight into potentially uncharted metabolic pathways. However, due to difficulties associated with metabolic profiling in this complex environment, metabolic changes during biofilm growth are still poorly characterized.

In the present study, we filtered through the wealth of molecules produced by mature *Escherichia coli* biofilms by restricting our analysis to comparison of volatile metabolites emitted from biofilm and planktonic cultures. We demonstrate that hypoxic conditions prevailing within biofilms induce high production of 1-propanol *via* a threonine fermentation pathway previously undocumented in *Enterobacteriaceae*, which confers a strong competitive advantage over bacteria unable to express this pathway. This study therefore demonstrates that investigation of metabolic changes associated with biofilm growth provides novel insights into the extent of bacterial metabolic potential and of bacterial adaptation to local microenvironments.

## Results

### Volatile 1-propanol is emitted by *E*. *coli* biofilms

**Fig 1:**
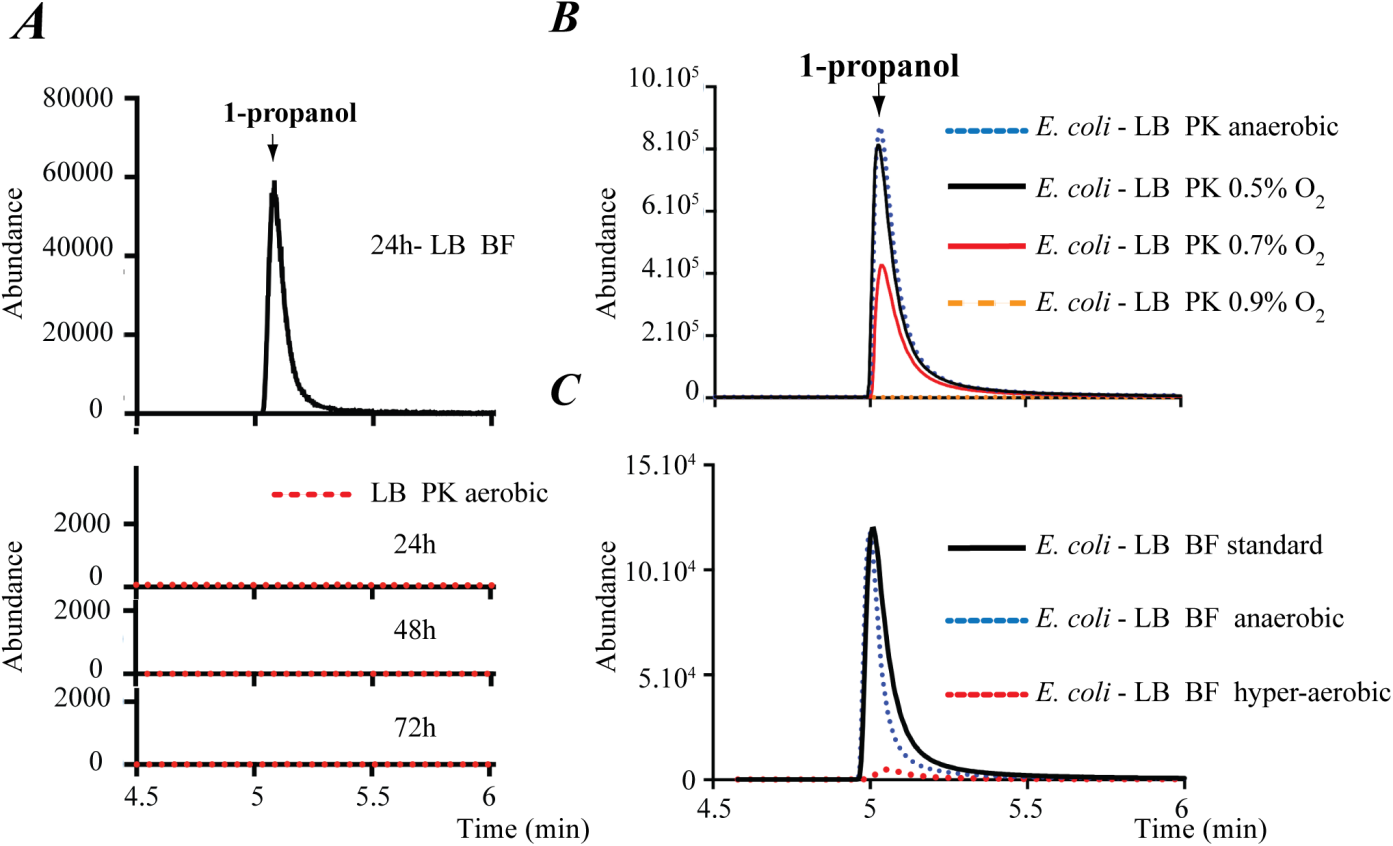
Influence of oxygen on 1-propanol production in *E*. *coli* cultures. (**A**) Comparison of HS-SPME/GC-MS analysis of 1-propanol emitted by wild-type *E*. *coli* (K12 strain TG1) biofilm grown for 24 h in LB medium (defined as “standard” biofilm conditions in Materials and Methods), or in agitated aerobic planktonic conditions for 24, 48 and 72 h. (**B**) HS-SPME/GC-MS analysis of 1-propanol emitted from *E*. *coli* planktonic cultures performed in LB medium under fully anaerobic or increasingly aerobic conditions. (**C**) HS-SPME/GC-MS analysis of 1-propanol emitted by *E*. *coli* biofilms formed in LB medium in standard, fully anaerobic or hyperaerobic conditions. Abundance: arbitrary unit proportional to the number of detected m/z ionized fragments.

We combined headspace solid-phase microextraction (HS-SPME) with gas chromatography mass spectrometry (GC-MS) to analyze volatile compounds emitted by *E*. *coli* biofilms formed in microfermenters. Under these conditions, bacteria growing at the periphery of biofilms are exposed to oxygen present in the medium, while inner biofilm bacteria are exposed to a range of microaerobic to anaerobic conditions. We then compared volatile compounds detected in biofilms to those produced by planktonic bacteria grown aerobically (S1 Fig). While these analyses revealed several differences, the most striking HS-SPME/GC-MS signal present in biofilm but absent in planktonic conditions corresponded to the volatile compound 1-propanol (Fig 1*A*). Although low level of 1-propanol is produced by some *Clostridium* strains [7], *E*. *coli* is not known to naturally produce this molecule, we therefore hypothesized the existence of a new *E*. *coli* metabolic pathway activated under conditions created by biofilm growth.

### Low oxygen conditions play a key role in 1-propanol production

Lack of 1-propanol production in aerobically grown planktonic cultures suggested that low oxygen conditions prevailing in biofilms might play a key role in *E*. *coli* 1-propanol production. Consistently, we observed 1-propanol signals in planktonic cultures grown in LB medium under fully anaerobic and microaerobic conditions, provided that the oxygen concentration did not exceed 0.7% (Fig 1*B*). Conversely, when biofilms were formed in highly aerobic biofilm microfermenters, we did not detect 1-propanol (Fig 1C). These results indicated that hypoxic conditions prevailing inside biofilms lead to 1-propanol production. Consistently, we showed that a mutation in the *fnr* gene encoding the primary transcriptional regulator mediating transition from aerobic to anaerobic growth [8] strongly reduced 1-propanol production (S2 Fig).

### The alcohol dehydrogenase AdhE is required for 1-propanol synthesis

**Fig 2:**
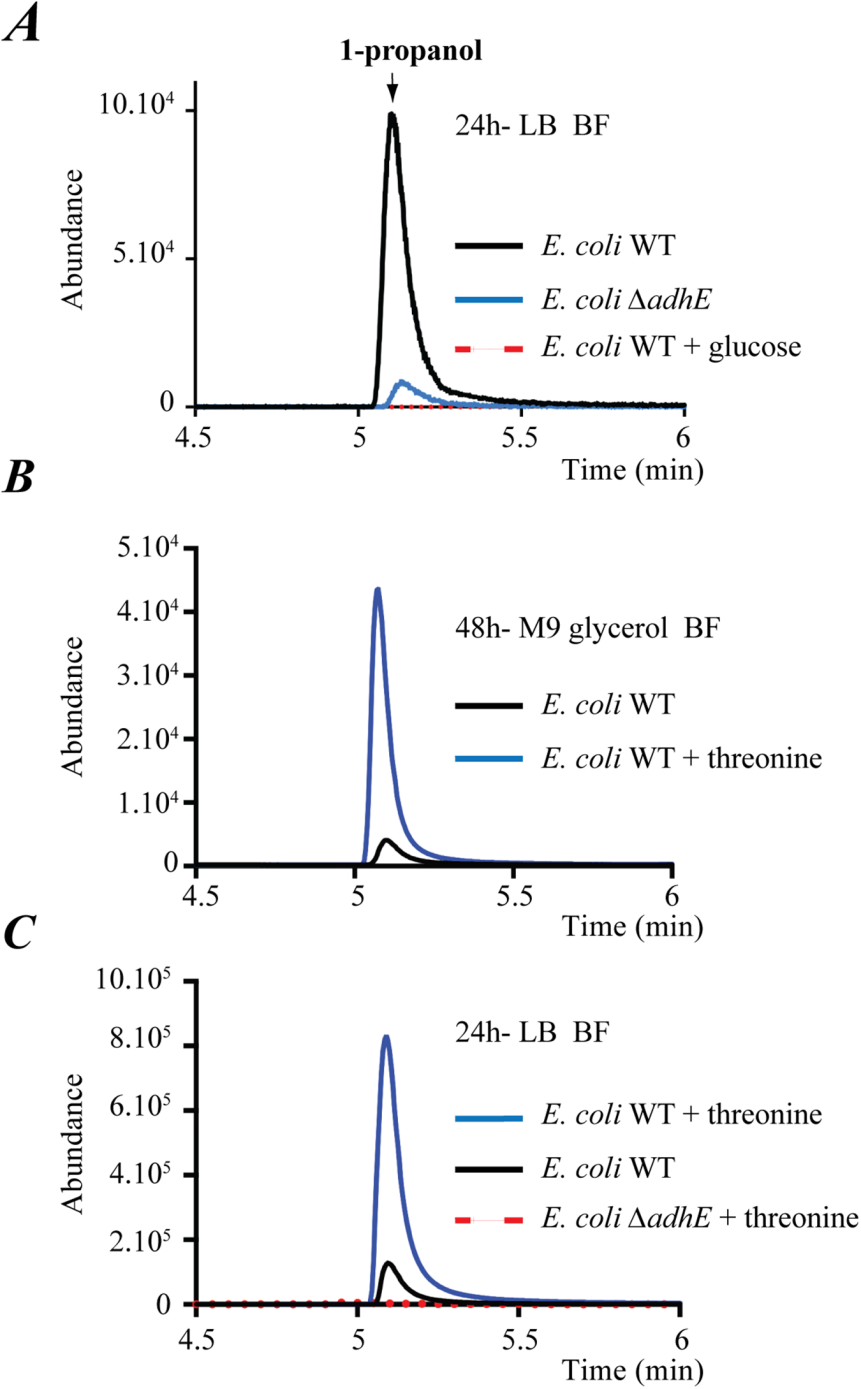
*E*. *coli* production of 1-propanol is AdhE-dependent and uses threonine as a precursor. (**A**) Comparison of HS-SPME/GC-MS analysis of 1-propanol emitted by *E*. *coli* WT and Δ*adhE* mutant biofilm grown for 24 h in LB medium under standard biofilm conditions (see also S2 Fig). (**B**) HS-SPME/GC-MS analysis of 1-propanol emitted from *E*. *coli* WT biofilms formed after 48 h in M9 glycerol minimal medium with or without 0.4% threonine. (**C**) HS-SPME/GC-MS analysis of 1-propanol emitted from *E*. *coli* WT and Δ*adhE* mutant biofilms formed in LB medium with or without 0.4% threonine.

Engineering approaches used to induce 1-propanol production in *E*. *coli* often rely on the expression of heterologous alcohol dehydrogenases from various microbial sources [7, 9]. We therefore hypothesized that at least one of the endogenous alcohol dehydrogenases of *E*. *coli* could contribute to 1-propanol production in biofilm. We tested the role of *E*. *coli* FucO, EutG, AdhE, YqhD and GlpQ alcohol dehydrogenases and determined that a mutation in the gene *adhE*, encoding a polymeric enzyme involved in ethanol production in *E*. *coli* and induced in anaerobic conditions [10] [11], resulted in a marked reduction in 1-propanol production (Fig 2*A* and S3 Fig). Lack of 1-propanol production observed in *E*. *coli ΔadhE* mutant could be restored in biofilm formed by *E*. *coli ΔadhE* complemented by pCAN*adhE* (S4 Fig).

### Threonine is a precursor of the 1-propanol pathway

**Fig 3:**
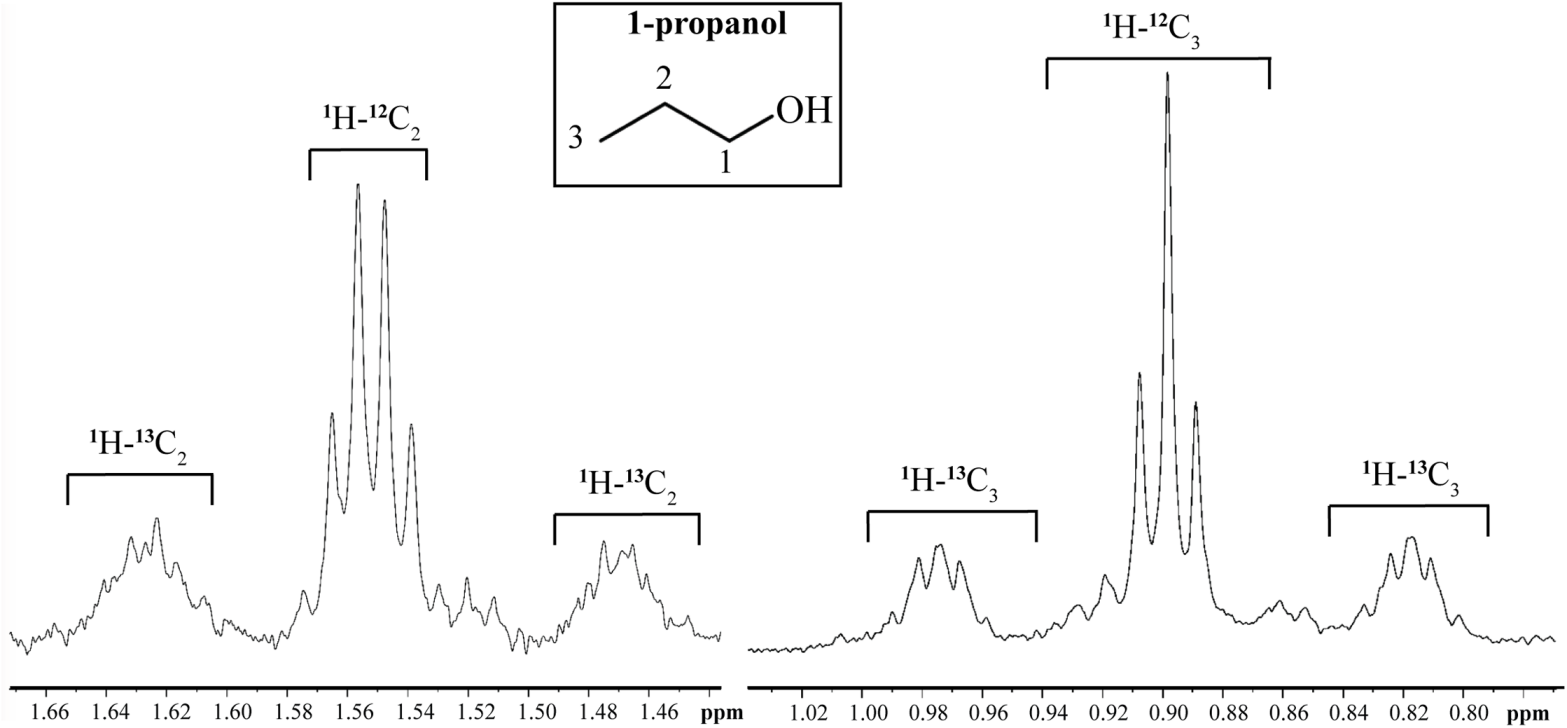
Incorporation of ^13^C into 1-propanol from [U-^13^C] L-threonine. *E*. *coli* wild-type biofilm was grown in M9 glycerol minimal medium supplemented with a mixture of unlabeled L-threonine and [U-^13^C]L-threonine (^13^C-enrichment was 44.5 ± 0.4%). Percent ^13^C incorporated into the carbon position is measured by the area of the ^1^H-^13^C signal relative to the total resonance area. Specific ^13^C-enrichments on C_2_ (41.4% ± 0.6%) and C_3_ (42.4% ± 0.2%) indicate the absence of significant isotopic dilution from ^13^C –labeled threonine when converted into 1-propanol. Insert: carbon positions in 1-propanol.

We next aimed to identify metabolic pathway constituents and necessary carbon sources for 1-propanol production. We observed that no 1-propanol could be detected in biofilms formed in LB supplemented with glucose (Fig 2*A*), indicating that 1-propanol production is subjected to catabolic repression, nor in biofilm formed in M9 glycerol minimal media (Fig 2*B*). However, supplementation of this medium with amino acids showed that L-threonine (threonine), but not valine, serine or glycine, triggered 1-propanol production (Fig 2*B*), as confirmed by NMR analysis (S5 Fig). Consistently, growth in LB medium containing 0.4% threonine led to a highly significant AdhE-dependent increase in 1-propanol production (Fig 2*C*). Finally, we monitored the steady-state level of incorporation of exogenous ^13^C-labeled threonine into 1-propanol in biofilm formed in M9 glycerol minimal medium. We observed strong isotope incorporation into 1-propanol, while we did not detect any significant isotope dilution by other unlabeled carbon sources (Fig 3). This demonstrated that all 1-propanol produced under these conditions originated from threonine degradation, which confirmed that threonine is the precursor of the 1-propanol pathway.

### Quantification of 1-propanol production in biofilm

Our results showed that 1-propanol can be directly produced in non-genetically-modified *E*. *coli* in a natural hypoxic microenvironment generated during biofilm growth. We observed that extension of biofilm cultures from 16 h to 48 h in biofilm microfermenters fueled with glycerol minimal medium supplemented with 0.4% threonine led to an increasingly strong 1-propanol signal (S6A Fig). This suggested that increasing biofilm biomass correlated with increased 1-propanol production. Moreover, the 1-propanol signal produced by 24 h biofilm strongly increased when using amino-acid-rich media such as TB, a medium containing 4 times more yeast extract than LB (S6B Fig). These results suggest that 1-propanol production could increase after prolonged biofilm growth in threonine-rich medium. Consistently, quantification of the amount of 1-propanol accumulated in the effluent of 48 h biofilm grown in TB or in TB supplemented with 0.4% threonine increased from 1.25 ± 0.15 g/L to up to 4.5 ± 0.34 g/L. These results therefore indicate that the 1-propanol yield could be optimized using amino-acid-rich unrefined media and *E*. *coli* biofilms as a production platform.

### 1-propanol is produced by via a TdcBE- and AdhE-dependent threonine degradation pathway

**Fig 4:**
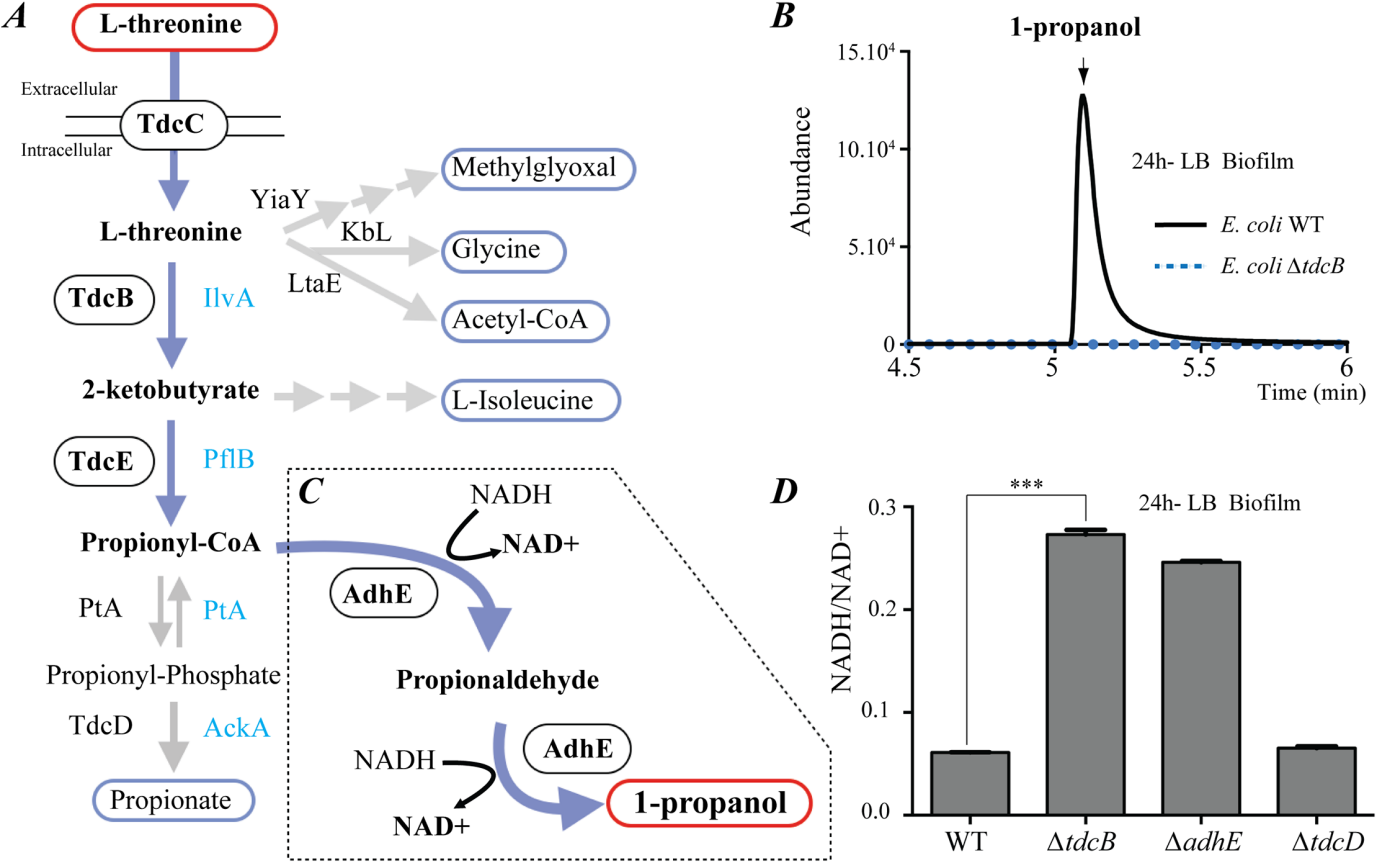
The TdcB- and AdhE-dependent 1-propanol pathway impacts *E*. *coli* redox balance. **(A)** Known *E*. *coli* threonine degradation pathways and their end products. Enzymes involved in the first steps of these pathways are indicated. Aerobic (blue) and anaerobic (black) threonine to propionate degradation pathway. Circled enzymes are essential for WT 1-propanol production. Due to its limited biofilm capacity, ability to produce 1-propanol of the Δ*tdcE* mutant was only tested in anaerobic planktonic conditions. (**B**) HS-SPME/GC-MS analysis of 1-propanol emitted from *E*. *coli* WT and Δ*tdcB* mutant biofilms formed in LB medium. (**C**) Dotted box: new *E*. *coli adhE*-dependent fermentation pathway of threonine into 1-propanol. NAD: nicotinamide adenine dinucleotide. NADH: reduced NAD; NAD^+^: oxidized NAD. (**D**) NADH/NAD^+^ ratios for *E*. *coli* WT, Δ*tdcB*, Δ*adhE* and Δ*tdcD* mutants grown under biofilm conditions. Error bars represent standard deviations from triplicate samples.

In *E*. *coli*, threonine has been shown to degrade into the end products acetyl-CoA, glycine, propionate, L-isoleucine and methylglyoxal, but not 1-propanol (Fig 4*A*). However, inactivation of genes involved in the first step of the known *E*. *coli* threonine degradation pathways, *ltaE*, *yiaY*, *kbL*, *ilvA* and *tdcB*, showed that only strains harboring a mutation in the threonine dehydratase gene *tdcB* impaired 1-propanol production in biofilm (Fig 4*B* and S1 Table). The lack of 1-propanol production observed in *E*. *coli ΔtdcB* mutant could be restored in biofilm formed by *E*. *coli ΔtdcB* complemented by pCAN*tdcB* (S4 Fig).

*tdcB* is part of the *tdc* operon, which is negatively regulated by catabolic repression and is involved in anaerobic uptake and degradation of threonine to propionate (Fig 4*A*) [12]. We tested the contribution of the other genes coding for enzymes involved in aerobic (*pflB*, *ptA* and *ackA*) and anaerobic (*tdcC*, *tdcE*, *ptA* and *tdcD*) propionate production, and showed that their inactivation did not affect 1-propanol production, except for *tdcC* (encoding a threonine uptake transporter) and *tdcE* (encoding a 2-ketobutyrate formate-lyase) (S1 Table and S7*A* and S7*B* Fig).

QRT-PCR gene expression analysis confirmed the strong induction of *tdcBDE* genes in planktonic anaerobic conditions (1,500-fold) and biofilms (300-fold) grown in LB medium (S8*A* and S8*B* Fig). AdhE catalyzes successive reduction of acetyl-CoA to acetaldehyde and the latter compound to ethanol [11]. We hypothesized that the promiscuous AdhE enzyme, which is also induced in biofilm conditions (S8*C Fig*), could carry out successive reduction of propionyl-CoA into propionaldehyde (coenzyme-A-dependent aldehyde dehydrogenase activity of AdhE), and then, reduction of propionaldehyde into 1-propanol (alcohol dehydrogenase activity of AdhE) (Fig 4*C*). Consistent with the existence, in *E*. *coli*, of a metabolic pathway branching out from the propionate pathway at the level of propionyl-CoA, we observed AdhE-dependent, increased production of 1-propanol in biofilms upon supplementation of LB medium with propionaldehyde (S9*A* Fig). We also observed concomitant increased production of ethanol in a *tdcB* mutant (S9*B* Fig), suggesting that blocking conversion of threonine into 1-propanol redirects AdhE metabolic activity towards ethanol synthesis.

### 1-propanol production in biofilm is widespread in *Enterobacteriaceae*

**Fig 5:**
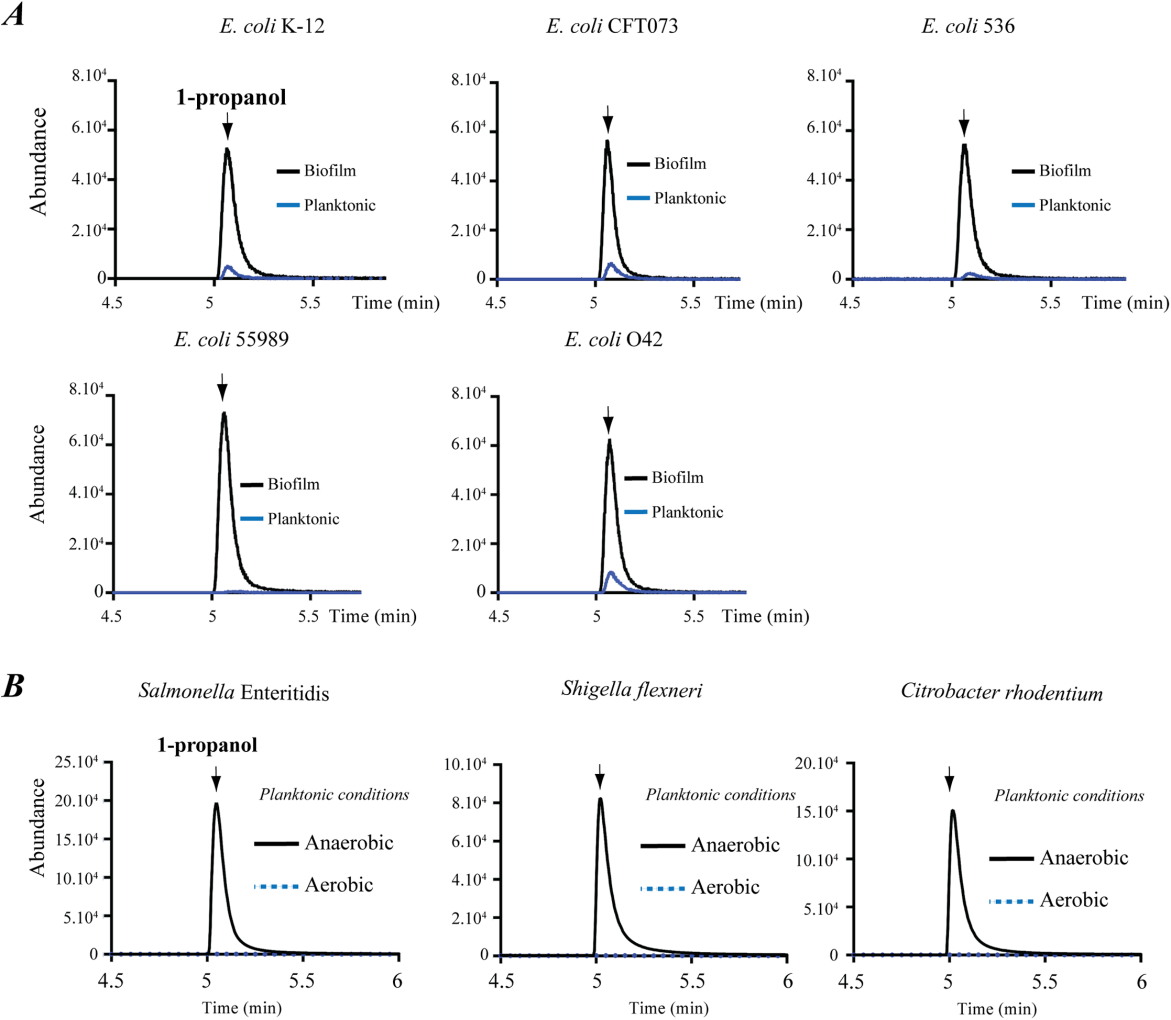
1-Propanol production in *E*. *coli* and other *Enterobacteriaceae*. **(A**) Detection of specific production of 1-propanol in biofilms formed by commensal *E*. *coli* K-12 and pathogenic *E*. *coli* isolates. Comparison of HS-SPME/GC-MS analysis of 1-propanol emitted by *E*. *coli* biofilm or planktonic bacterial biomass grown in LB medium. Biofilm was grown for 24 h in biofilm microfermenters or in an agitated flask in aerobic planktonic conditions. **(B)** Detection of specific production of 1-propanol in the indicated *Enterobacteriaceae*. Comparison of HS-SPME/GC-MS analysis of 1-propanol emitted by planktonic bacterial biomass grown in LB medium in anaerobic or aerobic conditions. No HS-SPME/GC-MS 1-propanol signal was detected in anaerobic or aerobic cultures of the following bacteria: *Enterobacter cloacae*, *Proteus mirabilis*, *Klebsiella pneumoniae*, *Aeromonas hydrophila*. Abundance: arbitrary unit proportional to the number of detected m/z ionized fragments.

We next examined conservation of this newly uncovered threonine degradation pathway among bacterial taxa. We determined that co-occurrence of homologs of *adhE*, *tdcB* and *tdcE* genes required for threonine degradation into 1-propanol can be identified in many bacteria, with the strongest homology found in *Enterobacteriaceae* (S10 Fig). Consistently, all tested *E*. *coli* isolates naturally produced 1-propanol in biofilms, while several other *Enterobacteriaceae* species, including *Shigella flexneri*, *Salmonella enterica* sv. Enteritidis and *Citrobacter rodentium*, also produce 1-propanol in anaerobic, but not in aerobic planktonic cultures (Fig 5).

### Threonine reduction into 1-propanol corresponds to a new *E*. *coli* fermentation pathway

Since *E*. *coli* K-12 cannot use 1-propanol as a carbon source, and exposure to 1-propanol did not display a detectable phenotype (S11 Fig), what could be the function of this threonine degradation into 1-propanol? Considering that reduction of propionyl-CoA into 1-propanol involves two successive steps of re-oxidation of reduced nicotinamide adenine dinucleotide (NADH) into NAD^+^ (Fig 4*C*), we hypothesized that degradation of threonine into 1-propanol recycles NADH into NAD^+^, a key co-factor playing a major role in central metabolism [11]. Indeed, analysis of the NADH/NAD^+^ ratio under biofilm and planktonic anaerobic growth conditions showed that bacteria exhibit an increased NADH/NAD^+^ ratio in Δ*tdcB* and Δ*adhE* mutants, but not in Δ*tdcD* mutants (Fig 4D and S12*A*, S12*B* and S12*C* Fig). Hence, while production of 1-propanol from threonine was reported in some *Clostridia* strains, it also corresponds to a previously undescribed native fermentation pathway in *E*. *coli*, contributing to intracellular redox balance in conditions of energy starvation in the absence of oxygen.

### Threonine fermentation provides a strong fitness advantage in anaerobic microenvironments

**Fig 6:**
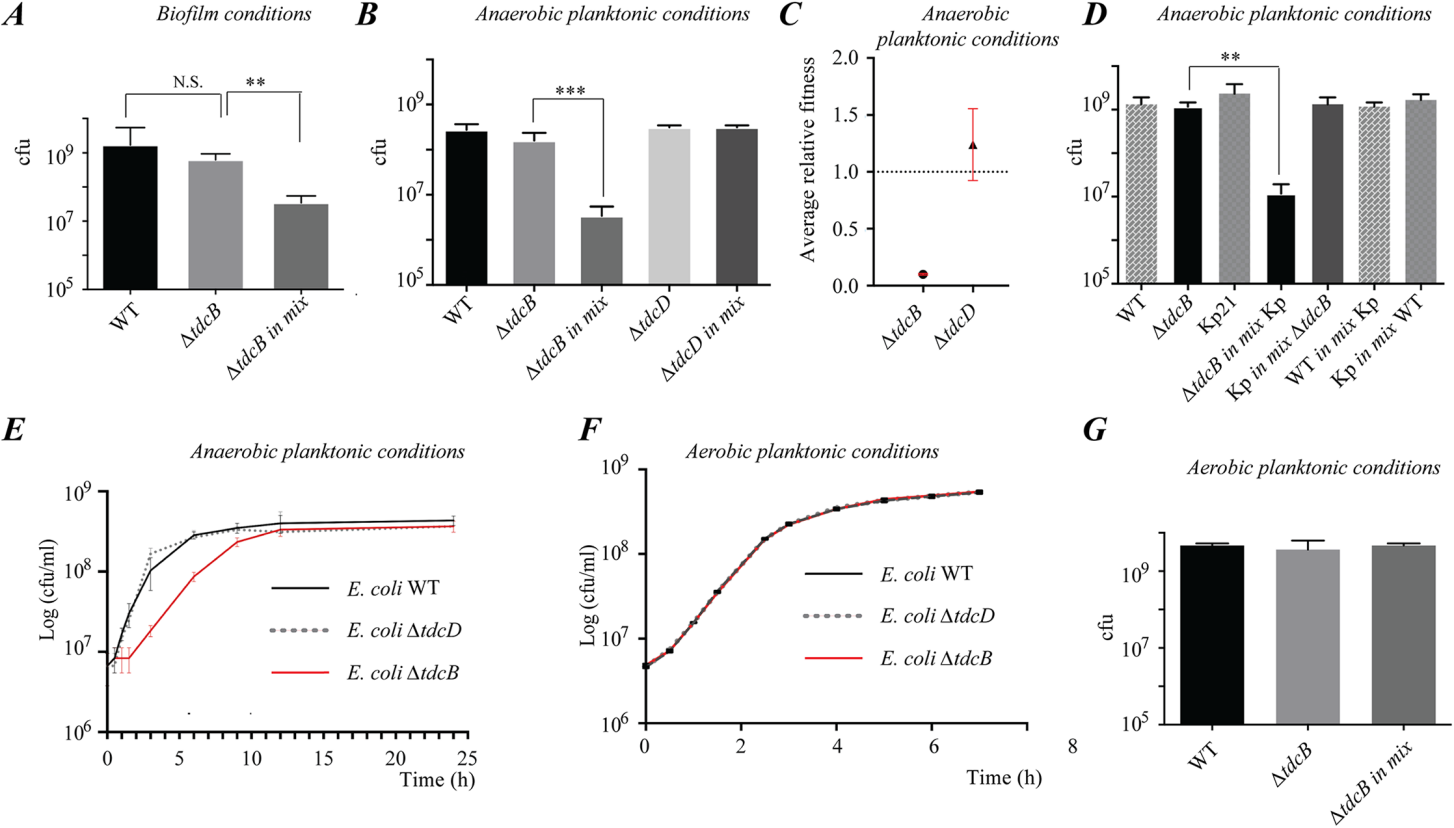
Impact of the *E*. *coli* 1-propanol pathway on fitness in biofilm or anaerobic planktonic competition experiments. (**A**) *E*. *coli* WT, and Δ*tdcB* mixed competition experiments performed in biofilm conditions. (**B**) *E*. *coli* WT, Δ*tdcB* and Δ*tdcD* mixed competition experiments performed in anaerobic planktonic conditions. (**C**) Comparison of fitness of WT, Δ*tdcB* and Δ*tdcD* mutants in mixed competition experiments performed in anaerobic planktonic conditions. Fitness was calculated as described in Materials and Methods. Dotted line represents WT fitness set to 1. (**D**) *E*. *coli* WT Δ*tdcB* and *Klebsiella pneumoniae* Kp21 mixed competition experiments performed in anaerobic planktonic conditions. (**E**) *E*. *coli ΔtdcB* displays an increased growth lag compared to WT or Δ*tdcD* mutant when grown in LB medium in anaerobic planktonic conditions. The recovery of Δ*tdcB* mutant after 15H is unlikely to be due to the accumulation of adaptive mutations, since *E*. *coli ΔtdcB* growth lag is consistently observed upon successive subcultures performed in triplicate in anaerobic planktonic conditions (S13 Fig). **(F)** Growth curves performed in aerobic planktonic conditions in LB medium, showing no lag or doubling time difference between *E*. *coli* WT, Δ*tdcB* or Δ*tdcD* mutants. **(G)** Mixed competition experiments performed in aerobic planktonic conditions, showing no decreased fitness of a Δ*tdcB* mutant compared to *E*. *coli* WT. Error bars represent standard deviations from triplicate samples.

To investigate the biological consequences of threonine fermentation in *E*. *coli*, we performed competition experiments between *E*. *coli* WT and a Δ*tdcB (no fermentation of threonine into 1-propanol)* or a Δ*tdcD* mutant (*threonine fermentation into 1-propanol*), either in biofilm or planktonic anaerobic conditions. Whereas the tested strains did not display any growth defect in monoculture (Fig 6*A* and 6*B*), we observed a 90% fitness reduction in the Δ*tdcB* mutant in competition experiments against WT when grown either in biofilm or planktonic anaerobic conditions (Fig 6*A*, 6*B* and 6*C*), whereas the *tdcD* mutant displayed no growth nor fitness defect (Fig 6*B*). Similarly, a Δ*tdcB* mutant display fitness reduction compared to WT, in competition experiments against *Klebsiella pneumoniae* (Fig 6*D*), a strain that does not ferment threonine into propanol. The fitness defect of a Δ*tdcB* mutant correlates with a consistent growth lag in planktonic anaerobic conditions compared to a wild-type or unaffected Δ*tdcD* mutant, (Fig 6*E* and S13 Fig). In contrast, a *tdcB* mutant did not display any growth lag (Fig 6*F*) or fitness cost when competition experiments were performed in aerobic planktonic culture conditions (Fig 6*G*), demonstrating the particular relevance of this novel pathway during anaerobic and biofilm growth. These results demonstrate that the threonine-to-propanol fermentation pathway contributes to provide a competitive advantage in mixed anaerobic communities.

## Discussion

We show that native production of 1-propanol is not restricted to a few anaerobic bacteria, but naturally occurs from threonine degradation under hypoxia in *E*. *coli* and other *Enterobacteriaceae*. The link between 1-propanol production and threonine catabolism was previously reported in *Clostridium* sp. strain 17cr1, which produces low amounts of 1-propanol (less than 70 mg/L) by an uncharacterized pathway [13]. Moreover, engineered *E*. *coli* strains can produce 1-propanol upon reduction of 2-keto-butyrate formed by the aerobic threonine degradation pathway [14]. In *E*. *coli*, we show here that anaerobic reduction of propionyl-CoA into propanal and 1-propanol by alcohol/aldehyde dehydrogenase AdhE constitutes a native alternative to anaerobic degradation of threonine into propionate and ATP synthesis by enzymes encoded by the *tdc* operon [12]. Although 1-propanol can be produced in anaerobic planktonic cultures, this metabolic capacity might have been overlooked due to the low biomass reached in these conditions. In contrast, we hypothesize that hypoxia spontaneously developing in biofilms enables a large bacterial biomass to be exposed to optimal conditions, inducing high production of 1-propanol.

1-propanol is an important industrial solvent and a major component of resins, the chemical synthesis of which requires a laborious two-step process involving catalytic hydroformylation of ethylene to produce propanal, and consecutive hydrogenation of propanal into 1-propanol [15–17]. Hence, improvement in renewable biological production of 1-propanol in metabolically engineered *E*. *coli* strains carrying heterologous genes of varying microbial origin recently gained significant attention [18, 19]. We determined that the yield of 1-propanol spontaneously produced in continuous flow biofilms in amino-acid-rich media could reach up to 5 g/L in the biofilm effluent, a yield close to levels obtained using engineered strains grown in discontinuous fed-batch cultures (<10 g/l) [7]. Achieving industrial productivity using bacterial biomass immobilization in biofilm reactors still presents many important obstacles and challenges [20]. However, our results suggest that bioproduction of 1-propanol from a native, and therefore robust, metabolic pathway induced in *E*. *coli* biofilms could alleviate the need for establishing synthetic pathways in genetically modified organisms, and might constitute an alternative to current 1-propanol chemical synthesis.

Fermentation is a central metabolic process that has been thoroughly investigated in *Enterobacteriaceae* in a variety of planktonic culture conditions, leading to production of lactate, ethanol, acetate, formate, citrate, succinate, hydrogen and carbon dioxide. However, our study reveals that, in addition to these classical fermentation products, *E*. *coli* and other *Enterobacteriaceae* can also produce 1-propanol upon reduction of threonine and reoxydation of NADH into NAD^+^, a staple of the fermentation process. The ability to ferment threonine as well as aromatic and branched-chain amino acids via the Stickland reaction is particularly used by anaerobic bacteria such as *Clostridia* [21–23]. This reaction is characterized by oxidation of one amino acid coupled with reduction of another amino acid [24]. However, we did not observe any stimulation of 1-propanol production upon supplementation with various amino acids, suggesting that threonine-to-propanol fermentation does not correspond to a *bona fide* Stickland reaction [25].

We show that reduction of threonine contributes to cellular redox balance by restoring the intracellular oxydized NAD^+^ pool, which may play an important role in *E*. *coli*’s ability to cope with anaerobic environments. Consistently, lack of threonine reduction into 1-propanol in a *tdcB* mutant leads to a remarkable 90% fitness loss in competition with the wild type strain. This decreased fitness could be attributed to a marked growth lag, enabling depletion of limited nutrient resources by the competing wild type strain. In the context of biofilm formation, maintaining redox balance is likely to be an essential metabolic process. Consistently, in absence of threonine fermentation, *E*. *coli* biofilm bacteria can use another fermentation pathway to recycle consumed NADH into NAD+, as shown by increased ethanol fermentation observed in a *tdcB* mutant (S9*B* Fig). However, these pathways generally rely on the availability of glucose or other oxidized sugars, which, unless produced by costly gluconeogenesis, may not be generally abundant in *E*. *coli* nutritional environment. By contrast, the amino-acid fermentation pathway described in this study could confer *Enterobacteriaceae* the ability to maintain redox balance and edge out competition with other bacteria, using amino-acids produced by cell lysis and proteolysis inside biofilms.

Another of such amino-acid rich environment could correspond to biofilm-like anaerobic gut environments, in which glycosylated mucins abundantly secreted in epithelial mucus contain up to 40% serine and threonine [26, 27]. However, *in vivo* mouse colonization competition experiments between *E*. *coli* WT and *tdcB* mutants did not reveal any significant colonization defect (S14 Fig), which might simply reflect the fact that the competitive fitness advantage provided by the propanol pathway cannot be relevantly tested in largely herbivorous mice.

Identification of a widespread metabolic response to biofilm and anaerobic conditions expands the range of known *E*. *coli* metabolites, opening perspectives of biofilm-based approaches for harnessing bacterial metabolic potential. Our study also further supports the notion that mining of the biofilm mode could provide insight into new aspects of bacterial physiological adaptations to local microenvironments.

## Materials and methods

### Bacterial strains and growth conditions

Bacterial strains used in this study are listed in S2 Table. *E*. *coli* mutants listed in S1 Table are from the Keio Collection [28] and each mutation was introduced into the *E*. *coli* TG1 strain by P1 *vir* phage transduction. Each mutant was confirmed by PCR. All experiments were performed in: lysogeny broth (LB) containing as amino acid sources 1% peptone and 0.5% yeast extract; Terrific broth (TB) containing 1.2% peptone, 2.4% yeast extract; or M9 glycerol 0.4% minimal medium containing no amino acid source. These media were supplemented with kanamycin (50 μg/ml) when required and incubated at 37°C. When needed, 0.4% (wt/vol) L-threonine (indicated as threonine throughout the text), glycine, serine or valine was added to the cultures. All media and chemicals were purchased from Sigma-Aldrich. Growth under anaerobic and microaerobic conditions was performed in a C400M Ruskinn anaerobic-microaerophilic station on multi-position magnetic stirrers XT35.1 (Roth Sochiel) at 37°C.

### Biofilm formation in microfermenters

Continuous-flow biofilm microfermenters containing a removable glass spatula were used as described in [6] (see also https://research.pasteur.fr/en/tool/biofilm-microfermenters/) in one of the following methods:

- Without internal bubbling of filter-sterilized compressed air (standard biofilm conditions). Under this condition, although microfermenters are fueled in aerobic conditions, lack of agitation favors rapid development of microaerobic to anaerobic conditions within the biofilm formed on the glass spatula inserted into the microfermenter.
- Without internal bubbling of a filter-sterilized compressed mix of 90% nitrogen / 5% hydrogen /5% carbon dioxyde (anaerobic biofilm conditions).
- With internal bubbling of filter-sterilized compressed air (hyperaerobic biofilm conditions).

Biofilm microfermenters were inoculated by placing the spatula in a culture solution adjusted to OD_600_ = 1 (containing 5.10^8^ bacteria/ml) for 5 min. The spatula was then reintroduced into the microfermenter. Flow rate was then adjusted (60 ml/h) so that total time for renewal of microfermenter medium was lower than bacterial generation time, thus minimizing planktonic growth by constant dilution of non-biofilm bacteria.

### Quantitative real-time PCR assays

Total RNA was extracted from three independent samples using the Qiagen RNeasy mini-kit. DNase treatment on 3 mg of RNA was carried out twice with the Ambion Turbo DNA-free kit. All samples were checked for residual genomic DNA contamination with the TM1 and TM2 primer pair (S3 Table), and were considered DNA-free if no amplification was detected at <38 cycles. The RNA concentration was measured with a NanoDrop and RNA quality was checked by gel electrophoresis. cDNA synthesis was carried out with 2 μg of RNA in a volume of 50 μl using the Bio-Rad iScript cDNA synthesis kit.

Primers for quantitative real-time PCR were designed using the Primer3 online tool (http://simgene.com/Primer3) and are listed in S3 Table. Amplicon sizes were confirmed by gel electrophoresis. cDNA levels were analyzed by EvaGreen detection in a Bio-Rad CFX96-1000 light cycler using the Bio-Rad SoFast EvaGreen Supermix (20 μl final volume), with 200 nM of each primer. Melting curves were checked to confirm that a single PCR product had been amplified. All quantitative real-time PCR reactions were carried out in quadruplicate for each sample in 96-well plates with simultaneous no-template controls. Relative quantification of gene expression levels was determined with the Delta Delta CT method [29] using 16S (*rssH*), *ihfb*, *opgG* and *hcaT* genes as reference genes.

### NMR analysis of 1-propanol released from *E*. *coli* biofilm

*E*. *coli* TG1 was grown in M9 glycerol 0.4% minimal medium supplemented with 0.2% L-threonine. The culture sample was collected after 48 h, centrifuged and the supernatant was frozen at -20°C prior to NMR analysis. 1D and 2D NMR spectra were recorded on a Bruker Avance III 800 MHz instrument (Bruker, Bremen, Germany), equipped with a 5 mm QPCI (^1^H, ^13^C, ^31^P and ^15^N) cryoprobe. Supernatants (120 μl) were mixed with 40 μl of 1 mM 2-(trimethylsilyl)propionic-2,2,3,3-d4 acid (TSP-d4) solution in D_2_O as an internal intensity and chemical shift standard, without further sample pretreatment. Data were acquired and processed using TOPSPIN 3.0 software.

### Isotope tracer experiments

*E*. *coli* biofilm was grown on M9 glycerol 0.4% minimal medium, supplemented with a mixture (1:1) of unlabeled L-threonine and [U-^13^C]L-threonine, in which the total concentration of L-threonine was 2 g/L. After 48 h of *E*. *coli* TG1 growth in the microfermenter at 37°C, the flux was stopped, and 15 h later, the biomass of the biofilm was recovered and centrifuged and the supernatant frozen until NMR analysis; Threonine ^13^C-enrichment was measured by NMR to be 44.5% ± 0.2%. The occurrence of a ^13^C atom resulted in splitting of ^1^H resonance due to ^1^H-^13^C coupling into ^1^H-^12^C (central peak) and ^1^H-^13^C (“satellite” peaks) signals. The percentage of ^13^C incorporated into the carbon position was measured by the area of ^1^H-^13^C signals relative to total resonance area. Specific ^13^C-enrichments on C2 (41.4% ± 0.6%) and C3 (42.4% ± 0.2%) of 1-propanol indicated the absence of significant isotopic dilution of ^13^C -labeled threonine when converted into 1-propanol. Insert: carbon positions in 1-propanol.

### Extraction and quantification of intracellular NADH and NAD^+^

Extraction of NADH and NAD^+^ was carried out according to the method described in [30]. Pellet samples of anaerobic planktonic culture or biomass of the biofilm were centrifuged at 16,000 x g for 1 min. Supernatant was removed and pellets were resuspended in 300 μl of 0.2M NaOH (for NADH extraction) or 0.2M HCl (for NAD^+^ extraction). These extracts were incubated for 10 min at 50°C and then for 10 min on ice. While vortexing, 300 μl of 0.1M HCl (for NADH) or 0.1M NaOH (for NAD^+^) was added dropwise to neutralize the solutions. Samples were then centrifuged for 5 min at 16,000 x g. Supernatants were transferred to fresh tubes and stored at -80°C until quantification.

Relative or absolute NADH or NAD^+^ levels were quantified using an enzyme cycling assay adapted for measurement in a microtiter plate [31]. A master reagent mix was prepared with 1 x bicine buffer (1.0 M pH 8), 3 x water, 1 x 40 mM EDTA, 1 x 100% ethanol, 1 x 4.2 mM thiazolyl blue and 2 x 16.6 mM phenazine ethosulfate. The reagent mix was warmed to 30°C, and then 90 μl aliquots were dispensed into individual wells of a 96-well microtiter plate; 5 μl of standard or sample were added to each well and the plate was incubated for approximately 10 min at 30°C. Then, the cycling reaction was started by the addition of 5 μl of alcohol dehydrogenase (Sigma no. A-3263) prepared at 347 units/ml in 0.1M bicine (pH8.0). The microtiter plate was incubated at 30°C, contents were mixed by brief shaking and absorbance was measured every 60 s using a TECAN Infinite M200 PRO at 570 nm, i.e. the spectral peak of thiazolyl blue that increases upon reduction.

Slopes arising from plots of absorbance at 570 nm over time were generated for NADH and NAD^+^ standards, as well as for all samples. Standard curves were used to calculate absolute concentrations in μM, and values were normalized to the optical density of the original cell culture sample.

### Fitness analysis: *In vitro* mixed culture competition experiments

#### Competition in anaerobic mixed cultures

Overnight planktonic anaerobic cultures of *E*. *coli* TG1 wild-type in LB medium were mixed at a 1:1 ratio (OD_600_ = 0.01) with overnight planktonic anaerobic cultures of *E*. *coli tdcB* or *tdcD* mutant before inoculation in LB medium, in an Erlenmeyer flask in an anoxic chamber at 37°C. As controls, individual monocultures of *E*. *coli* TG1 wild-type, *E*. *coli tdcB* and *tdcD* mutants were also prepared. The number of bacteria corresponding to each strain in the monoculture or the mixed cultures was evaluated by counting colony-forming-units (cfu) after serial dilution and plating at different time points on LB agar plates with suitable antibiotics. Fitness was calculated as follows: [cfu mutant in the mix after 24 h/(cfu wild-type in the mix after 24 h + cfu mutant in the mix after 24 h)]/proportion of the mutant in the mix at T_0_.

#### Competition in biofilm mixed cultures

Overnight cultures in LB medium of *E*. *coli* TG1 wild-type and *tdcB* or *tdcD* mutant were mixed at a 1:1 ratio (OD_600_ = 1) before inoculation in a biofilm microfermenter (LB medium 60 ml/h flow rate). Mixed biofilms were grown 24 h in LB medium before recovery and dilutions of the biomass were plated on LB with and without suitable antibiotics.

### *In vivo* mixed cultures competition experiments in mice

#### Ethic statement

All the described animal work was done in the Institut Pasteur animal facilities, which are accredited by the French Ministry of Agriculture to perform experiments on live rodents (accreditations A75-15 27, issued on 12 November 2004, and A75-15 04, issued on 22 May 2008). Work on animals was performed in compliance with French and European regulations on care and protection of laboratory animals (European Commission directive 2010/63; French law 2013–118, 06 February 2013). The protocols used in this study were approved by the ethics committee of the Institut Pasteur under the reference CETEA 2013–0064 and validated by the French ministry of Research under the reference MESR 00541. Reporting of our animal experiments follows the ARRIVE guidelines [32]. ***Methods*:** Female BALB/cAnNCrl mice (7 weeks) were purchased from Charles River Laboratories and housed in cages with *ad libitum* access to food. They were given sterile water containing 5 g/L of streptomycin sulfate 48 h before bacterial inoculation, and then throughout the experiment, to deplete gut aerobic microbiota. Sterile water was also supplemented with 0.8% threonine 5 days before bacterial inoculation and then throughout the experiment. 100 μl bacterial suspensions containing 10^5^ cfu (in PBS 1X) of either *E*. *coli* TG1 WT or its *tdcB* mutant were given intragastrically alone or in 1:1 mixed ration. Five mice were infected for each condition (wild-type strain alone, *tdcB* mutant strain alone and both strains in 1:1 mixed ratio). 24 h after inoculation, feces were collected, homogenized in PBS 1X, and serial dilutions were plated onto LB agar plate containing streptomycin (100 μg/mL) and appropriate antibiotics (kanamycin 50 μg/mL) when necessary. For determination of colonization in small intestine, cecum and large intestine, animals were sacrificed at day 1 post-inoculation by CO_2_ exposure. The potential impact of a *tdcB* mutation on colonization and/or fitness was assessed by comparing the number of cfu in feces, small intestine, cecum and large intestine from mice inoculated with the *tdcB* mutant alone or in 1:1 mixed ration with wild type, with the number of cfus of the wild-type strain in corresponding samples (alone or in 1:1 mixed ration). A P value of 0.05 was considered statistically significant.

### HS-SPME/GC-MS analysis of volatile compounds

Volatile compounds emitted by bacterial planktonic or biofilm cultures were determined using an analytical approach coupling headspace solid phase microextraction (HS-SPME) with gas chromatography and mass spectrometry (GC-MS) [33] [34].

Preparation of biofilm extracts for HS-SPME/GC-MS analysis was as follows. After 24 h of culture at 37°C, biofilm biomass that had formed on the spatula was scraped off, put in a 10 ml headspace vial and frozen until HS-SPME/GC-MS analysis (see S1 Fig).

1-Propanol was purchased from Sigma-Aldrich (Saint Quentin Fallavier, France). Ultra-pure water was produced using a Direct-Q UV 3 system (18.2 MΩ/cm) from Millipore (Molsheim, France); 75 μm carboxen-polydimethylsiloxane (CAR-PDMS) fiber was from Supelco (Sigma-Aldrich, Saint Quentin Fallavier, France) was used for SPME.

The fiber used was conditioned prior to performing analyses by inserting them into the GC injector at 280°C for 10 min. For each HS-SPME analysis, equivalent planktonic or biofilm bacterial biomass resuspended into 1 ml of planktonic or biofilm medium was introduced into 10 mL SPME vials. The fully automated HS-SPME procedure was as follows. First, the vial was equilibrated at 60°C for 6 min; then, the SPME fiber was placed into the head-space of the sample vial for extraction and maintained at 60°C for 30 min. At the end of extraction, the SPME fiber was introduced directly into the GC injector (desorption) for 10 min at 280°C in split mode (ratio1:2). GC-MS analyses were performed on an Agilent 7890A gas chromatograph coupled with an Agilent detector 5975C inert XL MSD mass spectrometer (Agilent Technologies, Les Ulis, France). The device is equipped with an MPS autosampler from Gerstel (RIC, Saint-Priest, France) that enabled fully automated HS-SPME analyses. The column used was a non-polar (methyl 95%-phenyl 5%) fused silica capillary column CP-SIL 8CB-MS (30 m x 0.25 mm with 1 μm film thickness) obtained from Agilent Technologies (Les Ulis, France). Helium was used as carrier gas in constant flow mode at 1 mL/min. The injector temperature was 280°C; injection mode was in split mode with a split ratio of 1:2. The temperature program was 40°C, held for 3 min and then raised to 60°C at 2°C/min, increased to 300°C at 20°C/min and held for 3 min (run 28 min). The transfer line temperature to the MS detector was set at 280°C. A mass spectrometer was used with the positive electronic ionization (EI) source (70 eV) heated to 230°C and the MS quad at 150°C. Acquisition was simultaneously performed in scan and SIM (single ion monitoring) modes. Scan acquisition was made from m/z 20 to 250. For SIM acquisition, the m/z fragments selected as characteristic fragment ions of 1-propanol were 31 (CH_2_ = OH^+^) and 59 (CH_3_-CH_2_-CH_2_O^+^). Identification of volatile organic compounds was performed by matching their recorded mass spectra with standard mass spectra from the National Institute of Standards and Technology (NIST, ver 2.0f, rev. 2010). The identity of each volatile compound was also confirmed by comparing their retention time and mass spectra with those of pure standard compounds after GC-MS analysis.

### Quantification of the 1-propanol concentration in biofilm and planktonic samples

The yield of 1-propanol in biofilm was determined either by resuspending 24 h biofilms formed on the internal microfermenter directly in microfermenter medium, or by resuspending 24 h biofilms for 15 or 24 h or 48 h after stopping medium flow, to allow accumulation of 1-propanol. The 1-propanol yield in anaerobic planktonic culture was tested by direct sampling of culture medium. After centrifugation, 20 ml of biofilm resuspension or planktonic culture supernatant were sent to Aromalyze (Quetigny, France http://www.aromalyse.fr). All samples were homogenized by vigorous stirring prior to analysis, and an aliquot of 0.5 ml was transferred into a 20 ml glass vial with a magnetic cap equipped with a PTFE/silicon septum containing 7.5 ml of deionized water and 2.5 g of NaCl (99%, Aldrich). As internal standard, an aqueous solution of 1-propanol-d7 (98atom% d, CDN Isotopes) at known concentration was added. All samples were analyzed by HS-SPME/GC-MS carried out at 40°C for 30 min using a carboxen-polydimethylsiloxane (CAR-PDMS) fiber (Supelco) on a CombiPAL. Fiber desorption was performed in the injector unit of the chromatograph in splitless mode. GC-MS analyses were carried out on a Shimadzu 2010 chromatograph coupled with a Shimadzu QP2010+ mass spectrometer. The capillary column used was an Rtx-624 (Restek) with stationary phase cyanopropylphenylated (6%) dimethyl polysiloxane (94%). The carrier gas was helium. Oven programming was as follows: 30°C for 5 min, 10°C/min to 150°C, 20°C/min to 300°C. Data acquisition was done in scan mode (m/z = 29 to 100, electron impact, 70eV). A blank sample (water replacing sample aliquot) was analyzed after each sample to exclude cross-contamination. All samples were analyzed in duplicate; several arbitrarily chosen samples were analyzed as triplicates. In all cases, the coefficient of variation on the calculated 1-propanol content was < 1.5%. Quantification was done using a signal of 1-propanol-d7 (isotope dilution analysis) and ions 60 for 1-propanol and 67 for 1-propanol-d7.

### Statistical analysis

Paired or unpaired Student t-test analyses were performed using Prism 6.0 for Mac OS X (GraphPad Software, Inc.). Each experiment was performed at least 3 times. * p<0.05; ** p<0.01; *** p<0.001.

## Supporting information

S1 Fig Set-up to detect and compare volatile compounds emitted from mature biofilms and planktonic cultures. (A) Biofilm and planktonic culture conditions. (B) Set-up for HS-SPME-based detection of volatile compounds emitted by planktonic or biofilm culture (see Materials and Methods).
(TIF)

S2 Fig 1-propanol production is regulated by the aerobic to anaerobic transition regulator FNR. Comparison of HS-SPME/GC-MS analysis of 1-propanol emitted in LB medium by biofilm formed by *E*. *coli* WT or by a Δ*fnr* mutant. Abundance: arbitrary unit proportional to the number of detected m/z ionized fragments.
(TIF)

S3 Fig Biofilm production of 1-propanol is AdhE-dependent. Comparison of HS-SPME/GC-MS analysis of 1-propanol emitted by *E*. *coli* WT and several alcohol dehydrogenase deletion mutants (Δ) grown for 24 h in LB medium under standard biofilm conditions. Abundance: arbitrary unit proportional to the number of detected m/z ionized fragments.
(TIF)

S4 Fig Complementation of biofilm 1-propanol production in *adhE* and *tdcB* mutants. Comparison of HS-SPME/GC-MS analysis showing that defect of 1-propanol emission in biofilm formed in LB medium supplemented with Kanamycin (50μg/ml), chloramphenicol (50μg/ml) and 0.1mM IPTG. *E*. *coli* Δ*tdcB* mutant or *E*. *coli* Δ*adhE* mutant carrying the empty vector pCAN24N can be complemented in strain *E*. *coli* Δ*tdcB* pCAN*tdcB* (= JW3088 in [35]) or *E*. *coli* Δ*adhE* pCAN*adhE* (= JW1228 in [35]) expressing respectively, *tdcB* and *adhE* gene. Abundance: arbitrary unit proportional to the number of detected m/z ionized fragments.
(TIF)

S5 Fig NMR identification of 1-propanol released from *E*. *coli* biofilm. Wild-type *E*. *coli* was grown in M9 glycerol minimal medium supplemented with 0.2% threonine. (A) Expansion of an 800 MHz 1D ^1^H NMR spectrum of culture medium showing the multiplet and triplet corresponding to Hβ and Hγ protons of 1-propanol, respectively. (B) Region of 800 MHz 2D [^1^H - ^13^C]-HSQC spectra collected from the same sample where peaks corresponding to Cβ and Cγ are annotated. (C) Region of an 800 MHz 2D [^1^H-^1^H] TOCSY NMR spectrum showing the correlation of Hγ with both Hβ and Hα of 1-propanol. Insert: carbon and hydrogen positions in 1-propanol.
(TIF)

S6 Fig Optimization of biofilm-associated production of 1-propanol. (A) Increase in 1-propanol HS-SPME/GC-MS signal over time in biofilm cultures. (B) Biofilm on internal glass spatula inserted into biofilm microfermenters formed in M9 glycerol minimal medium with 0.4% threonine.after 16, 24 and 48 h of growth (C) Comparison of 1-propanol HS-SPME/GC-MS signal in 24 h biofilm culture performed in rich LB medium containing the following amino acid (and therefore threonine) sources: 1% peptone, 0.5% yeast extract, or richer Terrific broth (TB) medium containing 1.2% peptone, 2.4% yeast extract. Abundance: arbitrary unit proportional to number of detected m/z ionized fragments.
(TIF)

S7 Fig *tdcE* but not t*dcD* is involved in *E*. *coli* 1-propanol production. (**A**) Comparison of HS-SPME/GC-MS analysis of 1-propanol emitted by biofilm formed by *E*. *coli* wild-type (WT) or Δ*tdcC* mutant in LB medium (**B**) Comparison of HS-SPME/GC-MS analysis of 1-propanol emitted by *E*. *coli* anaerobic planktonic culture of the WT or Δ*tdcE* mutant in LB medium. Due to its limited adhesion capacity, the Δ*tdcE* mutant could not be meaningfully tested in biofilm, but did not display growth defects in planktonic growth conditions. Abundance: arbitrary unit proportional to the number of detected m/z ionized fragments.
(TIF)

S8 Fig *tdcBDE* genes are induced in anaerobic and biofilm conditions. (**A**) Induction (fold expression) of genes involved in threonine degradation in anaerobic compared to (normalized) aerobic planktonic conditions in LB medium. (**B**) Induction (fold expression) of genes involved in threonine degradation in biofilm compared to (normalized) planktonic aerobic conditions.(**C**) Induction (fold expression) of genes involved (*adhE*) or not involved (*pflB*) in *E*. *coli* 1-propanol production in biofilm compared to (normalized) planktonic aerobic conditions.
(TIF)

S9 Fig Evidence for propionyl-CoA to 1-propanol conversion in biofilm conditions. (**A**) Comparison of HS-SPME/GC-MS analysis of 1-propanol emitted by biofilm formed by *E*. *coli* wild-type (WT) or the Δ*adhE* mutant upon supplementation of LB medium with 0.02% propionaldehyde, showing *adhE*-dependent conversion of propionaldehyde into 1-propanol. AdhE-independent conversion of propionaldehyde into 1-propanol is likely due to other *E*. *coli* promiscuous alcohol dehydrogenases. (**B**) Comparison of HS-SPME/GC-MS analysis of ethanol produced by biofilm formed by *E*. *coli* WT or the Δ*tdcB* mutant in LB medium. Abundance: arbitrary unit proportional to number of detected m/z ionized fragments.
(TIF)

S10 Fig Co-occurrence of *adhE*, *tdcB* and *tdcE* in bacteria. Organisms with homologs of AdhE, TdcB and TdcE proteins encoded by *E*. *coli* K12 *adhE*, *tdcB* and *tdcE* genes. Presence or absence of these homologs in an organism is indicated by a quantitative color scale showing the extent of amino acid sequence conservation between AdhE, TdcB and TdcE proteins and their most-similar ortholog; white indicates 0% identity; dark red indicates 100% identity. Analysis used the STRING Search Tool [36].
(TIF)

S11 Fig Experimental set-ups used to study the potential biological role of 1-propanol. Using a variation of the 2-petri dish assay [37, 38], *E*. *coli* strain MG1655, *Pseudomonas aeruginosa* strain PAO1 and *Staphylococcus aureus* strain HG001 were assayed for growth, motility and biofilm formation upon exposure to volatile 1-propanol emitted from 0.1%, 0.5% and 1% solutions. No phenotypic differences were observed compared to unexposed bacteria.
Phenotypic tests used to identify a direct biological role for 1-propanol. The potential impact of exposure to volatile 1-propanol emitted from 0.1%, 0.5% and 1% solutions on growth, motility and biofilm formation was tested, as described in S10 Fig, on *E*. *coli* strain MG1655, *P*. *aeruginosa* strain PAO1 and *S*. *aureus* strain HG001. Motility was not tested for the non-motile *S*. *aureus* strain HG001 strain. No phenotypic differences could be observed compared to unexposed bacteria. The potential direct toxicity of 1-propanol was tested by supplementing 1-propanol in planktonic aerobic and anaerobic *E*. *coli* cultures (physiological concentration determined in anaerobic planktonic culture in LB medium is ca. 0.03% final). The presence of 0.01%, 0.1%, 0.5% and 1% 1-propanol (final concentration) had no detectable growth impact on *E*. *coli* strain TG1.
(TIF)

S12 Fig Impact of *E*. *coli* 1-propanol pathway on redox balance in biofilm and anaerobic planktonic conditions. (**A**) NADH and NAD^+^ concentrations for *E*. *coli* wild-type (WT), Δ*tdcB*, Δ*tdcD* and Δ*adhE* mutant cultures grown under biofilm conditions in LB medium. (**B**) NADH and NAD^+^ concentrations for *E*. *coli* WT, Δ*tdcB* and Δ*tdcD* mutants cultures grown under anaerobic planktonic conditions in LB medium. In these conditions, the Δ*adhE* mutant exhibits a strong growth defect and cannot be meaningfully tested. (**C**) Impact of *E*. *coli* 1-propanol pathway on redox balance in LB medium under anaerobic planktonic conditions. Error bars represent the standard deviations from triplicate samples.
(TIF)

S13 Fig *E*. *coli* Δ*tdcB* growth lag is consistently observed upon successive sub-cultivation. Comparison of *E*. *coli* Δ*tdcB* growth lag upon successive sub-cultivation show that *E*. *coli* Δ*tdcB* growth lag is consistently observed compared to wild type, indicating that Δ*tdcB* mutant recovery at 25H is not due accumulation of adaptive mutations. *S*uccessive subculture performed in triplicate in anaerobic planktonic conditions.
(TIF)

S14 Fig Impact of threonine fermentation into 1-propanol on mouse gut colonization and fitness *in vivo*. *E*. *coli* WT strain or *tdcB* mutant, alone or in mixed competition experiments performed *in vivo* show no significant decreased fitness of a *tdcB* mutant compared to *E*. *coli* WT. Colonization of small intestine by both *E*. *coli* strains is highly variable since 2 to 3 mice per conditions (10 mice in total) were not colonized in this section, so the corresponding values are not included on the graph.
(TIF)

S1 Table Complete list of *E*. *coli* K-12 mutants tested in this study and their impact on HS-SPME/GC-MS propanol signal. (PDF)

S2 Table List of strains used in this study. (PDF)

S3 Table Complete list of oligonucleotide primers used to perform qRT-PCR experiments in this study. (PDF)
