# Supplementary figures and images for: Biofilm microenvironment induces a widespread adaptive amino-acid fermentation pathway conferring strong fitness advantage in Escherichia coli
Source: PLoS Genet. 2017 May 19;13(5):e1006800. doi: 10.1371/journal.pgen.1006800 (PMC5459495; doi:10.1371/journal.pgen.1006800)

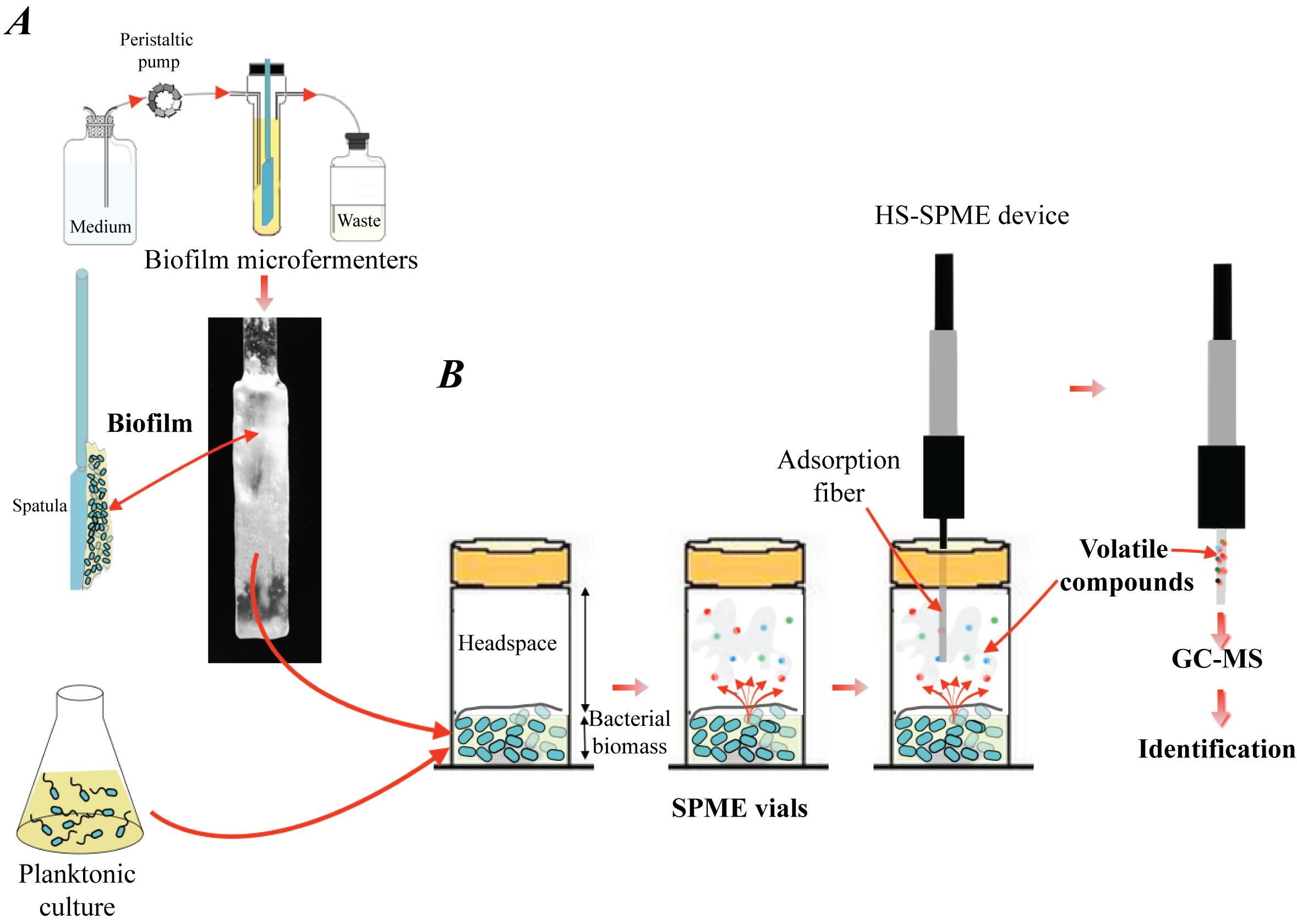

Supplement: S1 Fig — (A) Biofilm and planktonic culture conditions. (B) Set-up for HS-SPME-based detection of volatile compounds emitted by planktonic or biofilm culture (see Materials and Methods). (TIF) [file pgen.1006800.s001.tif]

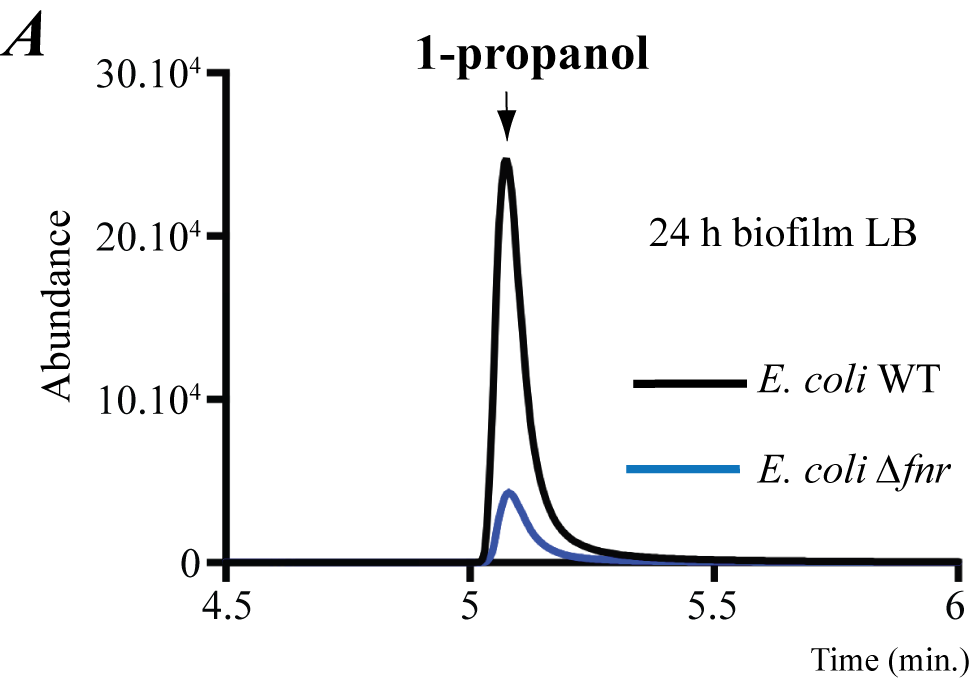

Supplement: S2 Fig — Comparison of HS-SPME/GC-MS analysis of 1-propanol emitted in LB medium by biofilm formed by E. coli WT or by a Δfnr mutant. Abundance: arbitrary unit proportional to the number of detected m/z ionized fragments. (TIF) [file pgen.1006800.s002.tif]

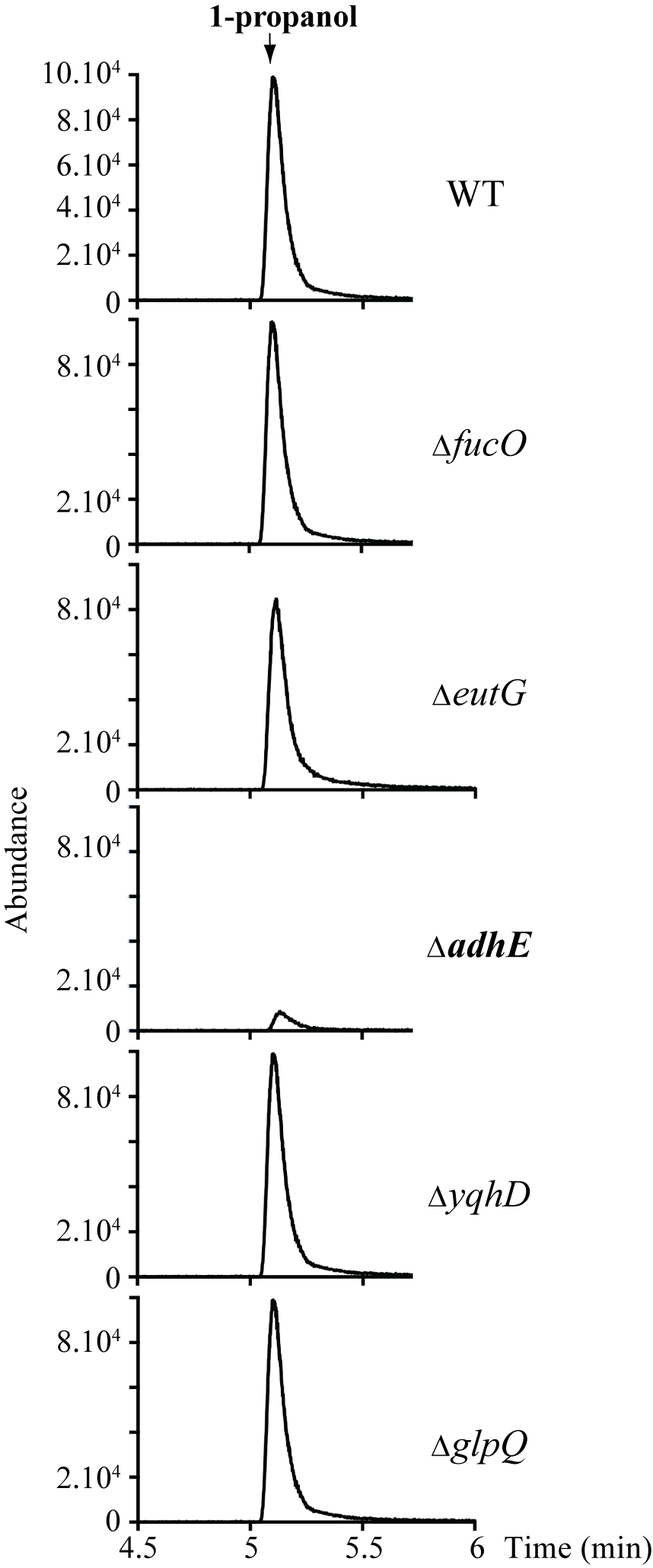

Supplement: S3 Fig — Comparison of HS-SPME/GC-MS analysis of 1-propanol emitted by E. coli WT and several alcohol dehydrogenase deletion mutants (Δ) grown for 24 h in LB medium under standard biofilm conditions. Abundance: arbitrary unit proportional to the number of detected m/z ionized fragments. (TIF) [file pgen.1006800.s003.tif]

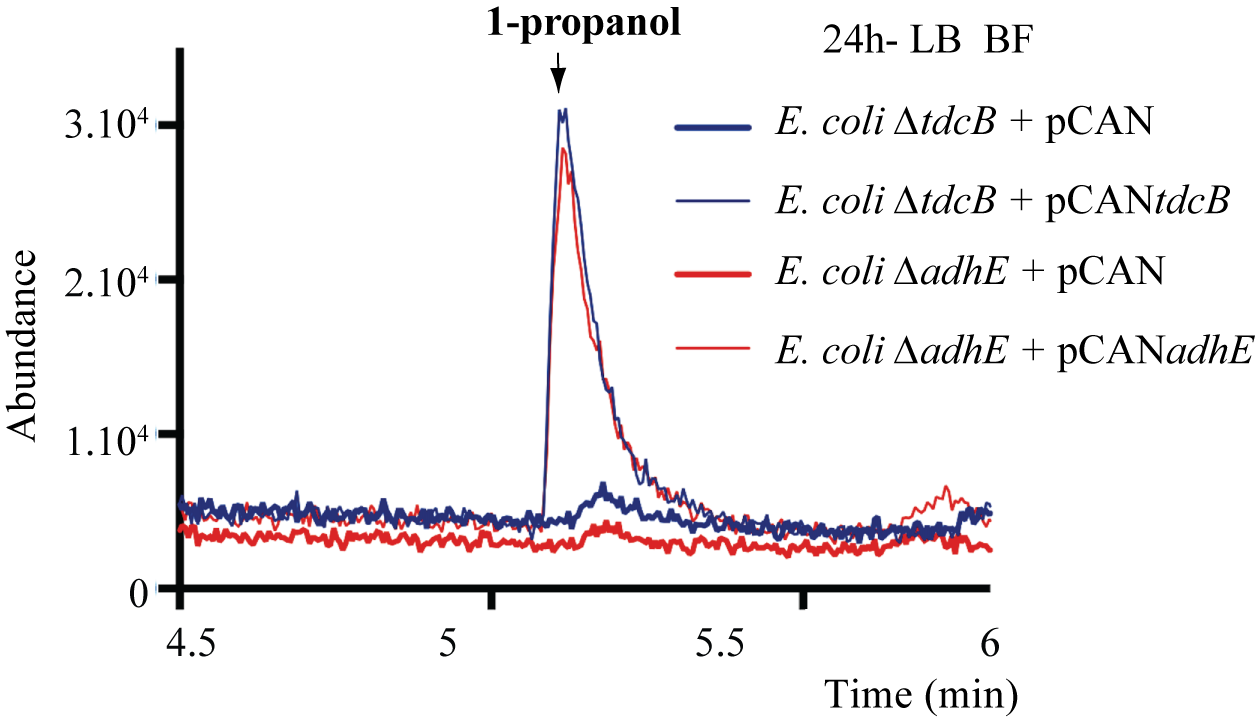

Supplement: S4 Fig — Comparison of HS-SPME/GC-MS analysis showing that defect of 1-propanol emission in biofilm formed in LB medium supplemented with Kanamycin (50μg/ml), chloramphenicol (50μg/ml) and 0.1mM IPTG. E. coli ΔtdcB mutant or E. coli ΔadhE mutant carrying the empty vector pCAN24N can be complemented in strain E. coli ΔtdcB pCANtdcB (= JW3088 in [35]) or E. coli ΔadhE pCANadhE (= JW1228 in [35]) expressing respectively, tdcB and adhE gene. Abundance: arbitrary unit proportional to the number of detected m/z ionized fragments. (TIF) [file pgen.1006800.s004.tif]

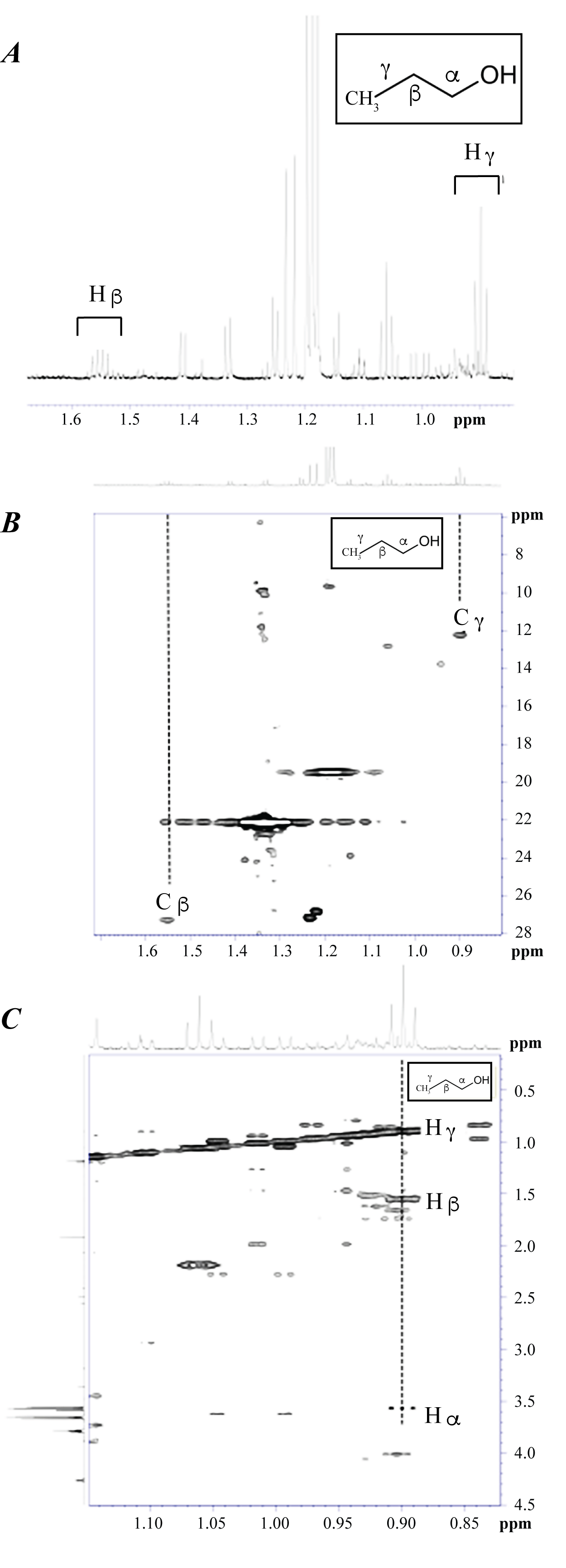

Supplement: S5 Fig — Wild-type E. coli was grown in M9 glycerol minimal medium supplemented with 0.2% threonine. (A) Expansion of an 800 MHz 1D 1H NMR spectrum of culture medium showing the multiplet and triplet corresponding to Hβ and Hγ protons of 1-propanol, respectively. (B) Region of 800 MHz 2D [1H - 13C]-HSQC spectra collected from the same sample where peaks corresponding to Cβ and Cγ are annotated. (C) Region of an 800 MHz 2D [1H-1H] TOCSY NMR spectrum showing the correlation of Hγ with both Hβ and Hα of 1-propanol. Insert: carbon and hydrogen positions in 1-propanol. (TIF) [file pgen.1006800.s005.tif]

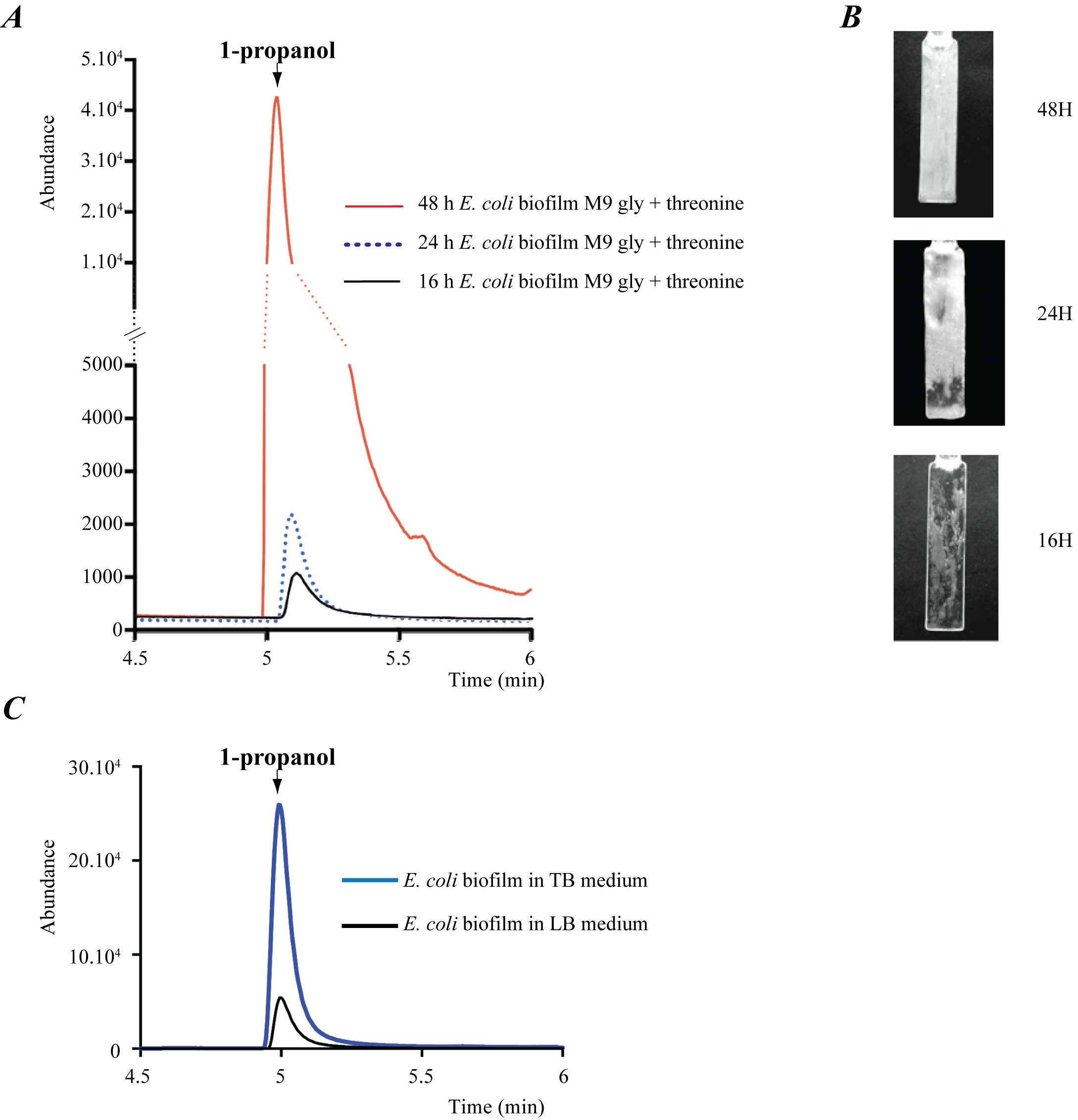

Supplement: S6 Fig — (A) Increase in 1-propanol HS-SPME/GC-MS signal over time in biofilm cultures. (B) Biofilm on internal glass spatula inserted into biofilm microfermenters formed in M9 glycerol minimal medium with 0.4% threonine.after 16, 24 and 48 h of growth (C) Comparison of 1-propanol HS-SPME/GC-MS signal in 24 h biofilm culture performed in rich LB medium containing the following amino acid (and therefore threonine) sources: 1% peptone, 0.5% yeast extract, or richer Terrific broth (TB) medium containing 1.2% peptone, 2.4% yeast extract. Abundance: arbitrary unit proportional to number of detected m/z ionized fragments. (TIF) [file pgen.1006800.s006.tif]

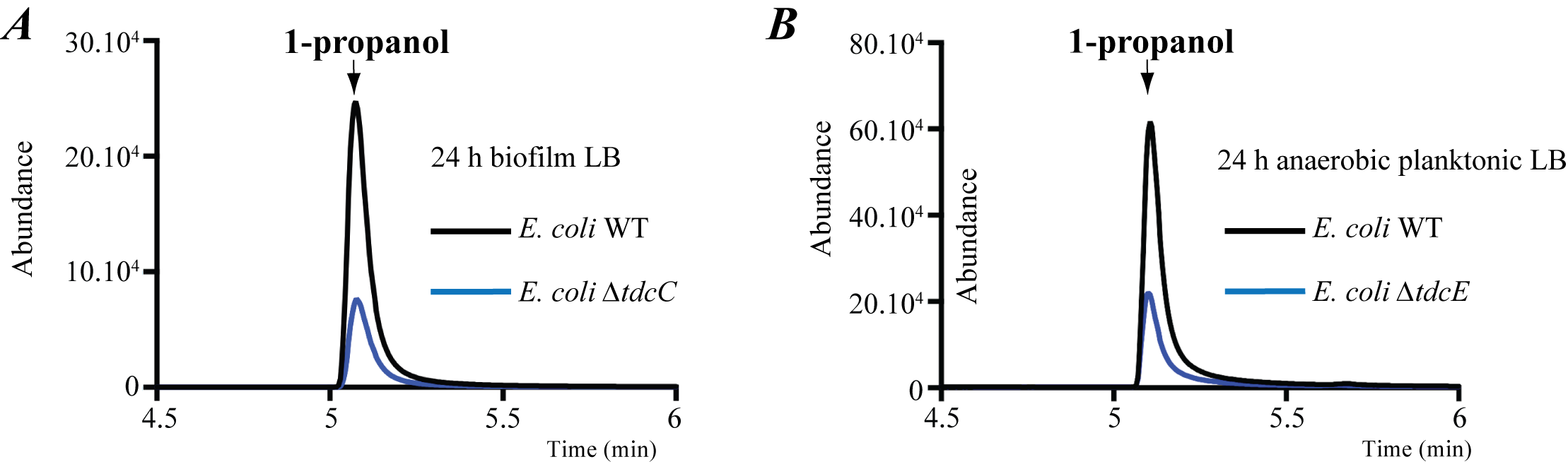

Supplement: S7 Fig — (A) Comparison of HS-SPME/GC-MS analysis of 1-propanol emitted by biofilm formed by E. coli wild-type (WT) or ΔtdcC mutant in LB medium (B) Comparison of HS-SPME/GC-MS analysis of 1-propanol emitted by E. coli anaerobic planktonic culture of the WT or ΔtdcE mutant in LB medium. Due to its limited adhesion capacity, the ΔtdcE mutant could not be meaningfully tested in biofilm, but did not display growth defects in planktonic growth conditions. Abundance: arbitrary unit proportional to the number of detected m/z ionized fragments. (TIF) [file pgen.1006800.s007.tif]

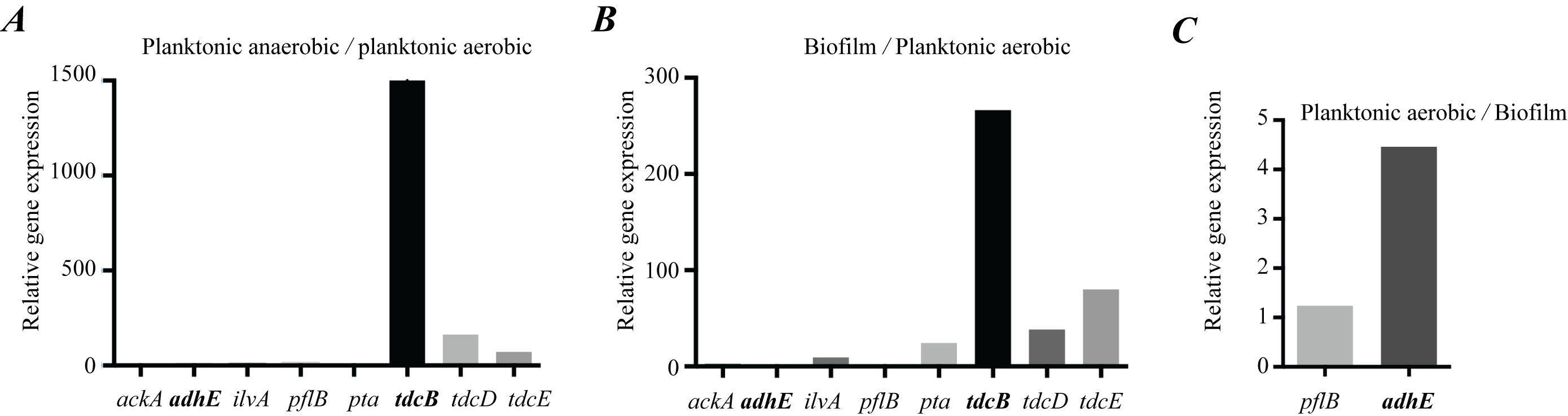

Supplement: S8 Fig — (A) Induction (fold expression) of genes involved in threonine degradation in anaerobic compared to (normalized) aerobic planktonic conditions in LB medium. (B) Induction (fold expression) of genes involved in threonine degradation in biofilm compared to (normalized) planktonic aerobic conditions.(C) Induction (fold expression) of genes involved (adhE) or not involved (pflB) in E. coli 1-propanol production in biofilm compared to (normalized) planktonic aerobic conditions. (TIF) [file pgen.1006800.s008.tif]

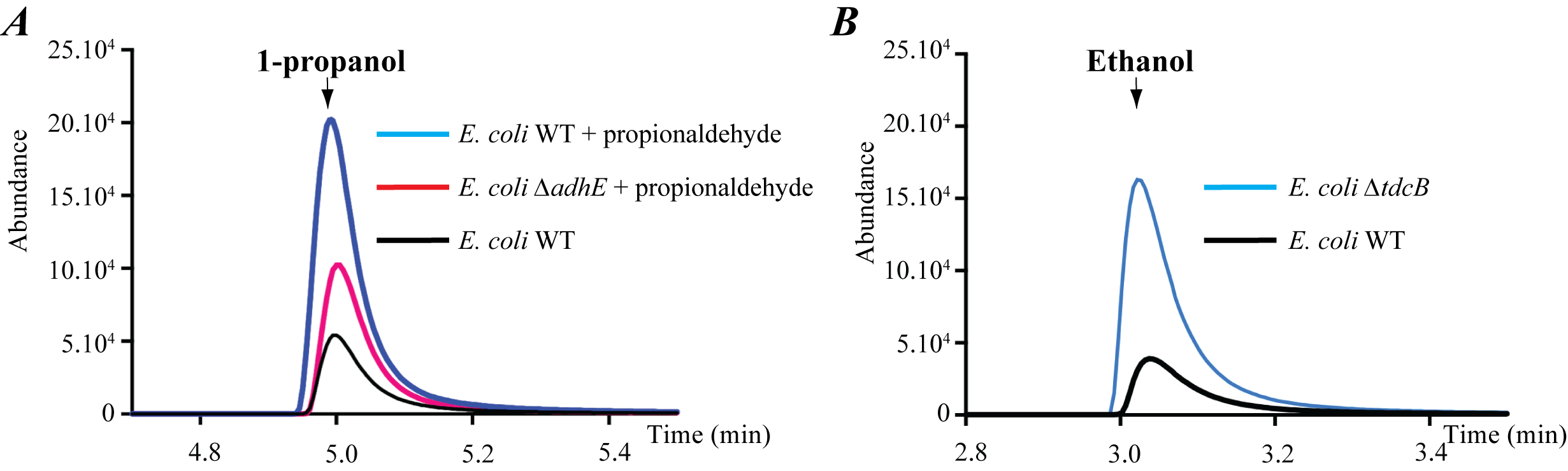

Supplement: S9 Fig — (A) Comparison of HS-SPME/GC-MS analysis of 1-propanol emitted by biofilm formed by E. coli wild-type (WT) or the ΔadhE mutant upon supplementation of LB medium with 0.02% propionaldehyde, showing adhE-dependent conversion of propionaldehyde into 1-propanol. AdhE-independent conversion of propionaldehyde into 1-propanol is likely due to other E. coli promiscuous alcohol dehydrogenases. (B) Comparison of HS-SPME/GC-MS analysis of ethanol produced by biofilm formed by E. coli WT or the ΔtdcB mutant in LB medium. Abundance: arbitrary unit proportional to number of detected m/z ionized fragments. (TIF) [file pgen.1006800.s009.tif]

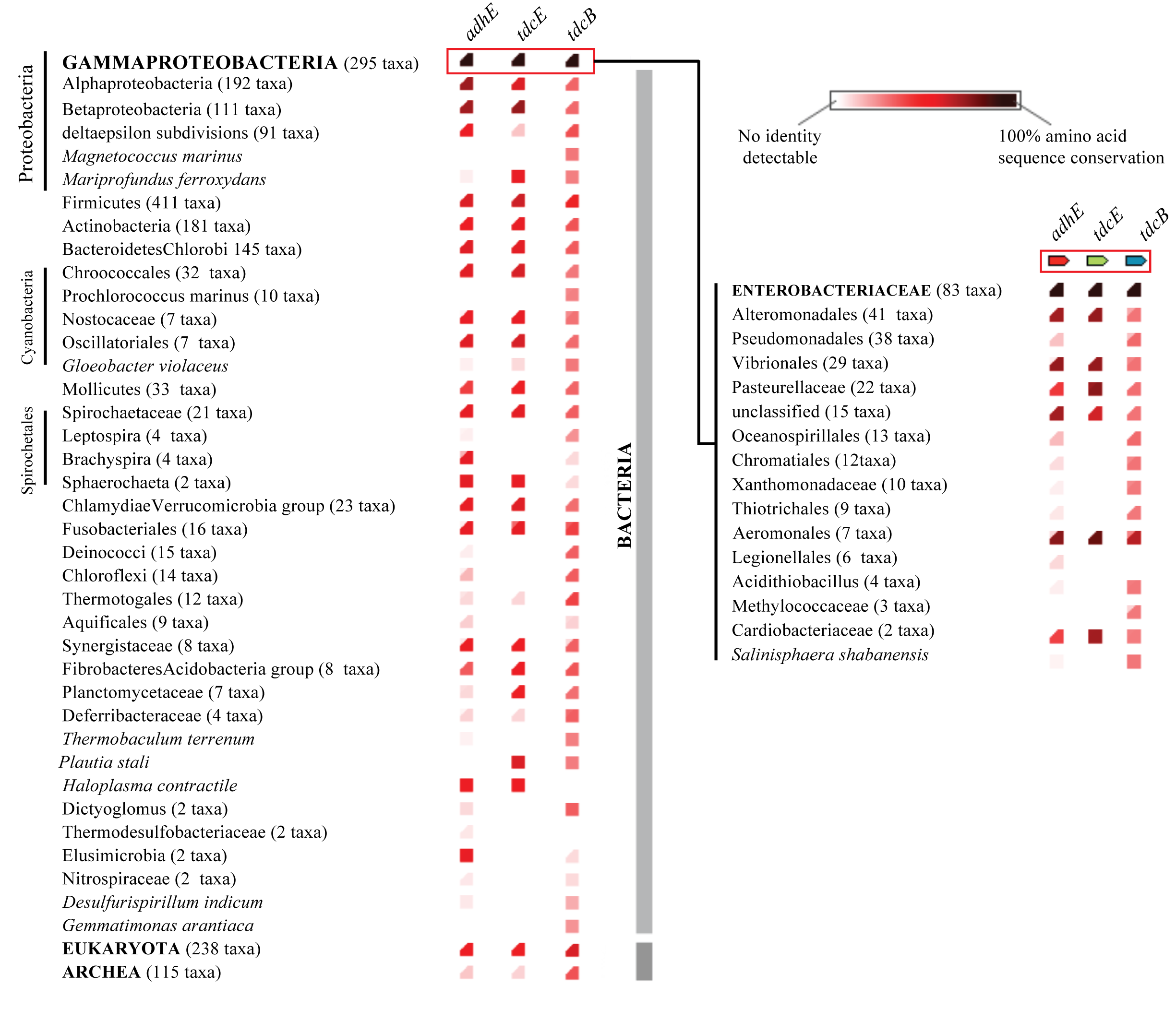

Supplement: S10 Fig — Organisms with homologs of AdhE, TdcB and TdcE proteins encoded by E. coli K12 adhE, tdcB and tdcE genes. Presence or absence of these homologs in an organism is indicated by a quantitative color scale showing the extent of amino acid sequence conservation between AdhE, TdcB and TdcE proteins and their most-similar ortholog; white indicates 0% identity; dark red indicates 100% identity. Analysis used the STRING Search Tool [36]. (TIF) [file pgen.1006800.s010.tif]

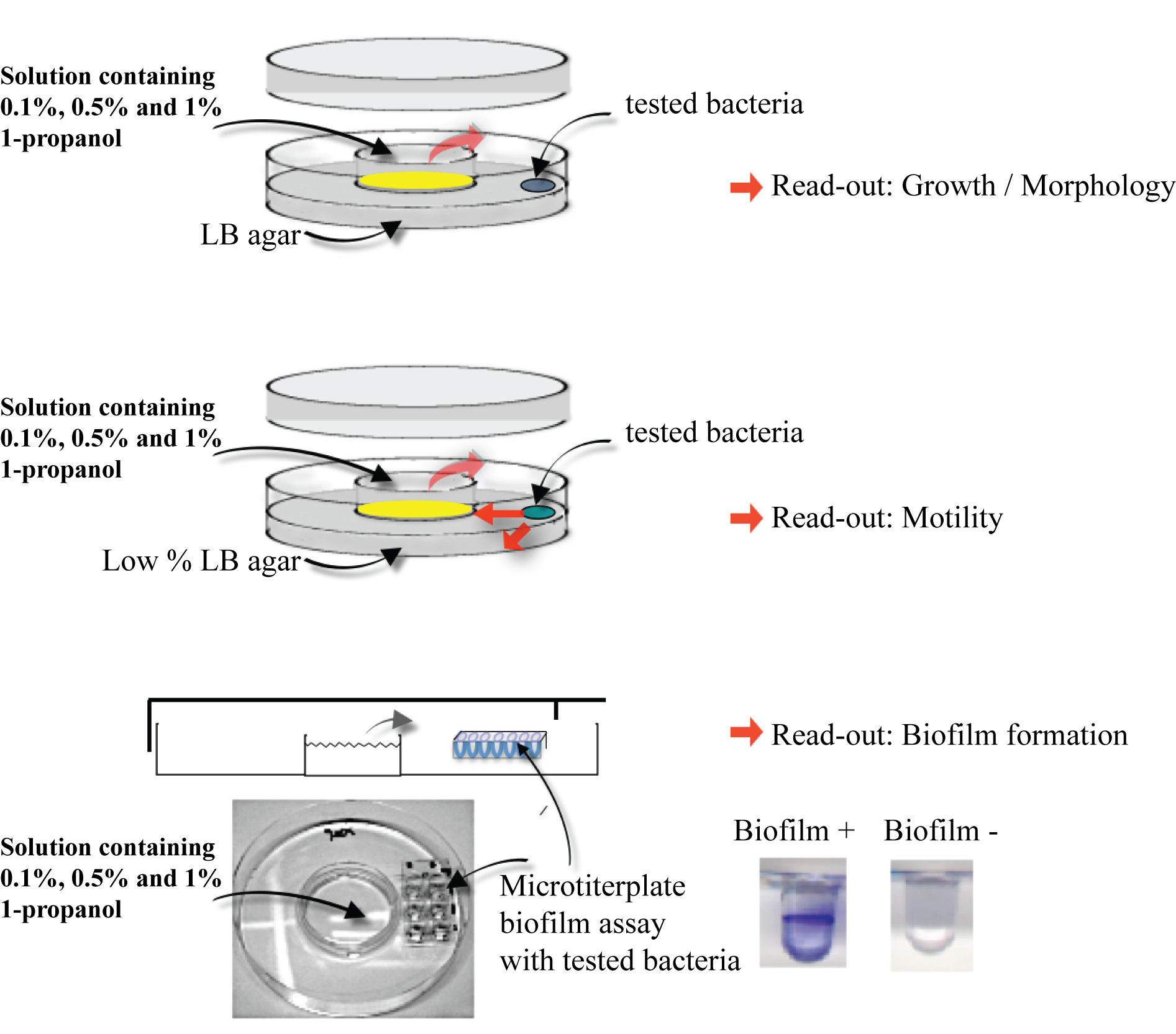

Supplement: S11 Fig — Using a variation of the 2-petri dish assay [37, 38], E. coli strain MG1655, Pseudomonas aeruginosa strain PAO1 and Staphylococcus aureus strain HG001 were assayed for growth, motility and biofilm formation upon exposure to volatile 1-propanol emitted from 0.1%, 0.5% and 1% solutions. No phenotypic differences were observed compared to unexposed bacteria. Phenotypic tests used to identify a direct biological role for 1-propanol. The potential impact of exposure to volatile 1-propanol emitted from 0.1%, 0.5% and 1% solutions on growth, motility and biofilm formation was tested, as described in S10 Fig, on E. coli strain MG1655, P. aeruginosa strain PAO1 and S. aureus strain HG001. Motility was not tested for the non-motile S. aureus strain HG001 strain. No phenotypic differences could be observed compared to unexposed bacteria. The potential direct toxicity of 1-propanol was tested by supplementing 1-propanol in planktonic aerobic and anaerobic E. coli cultures (physiological concentration determined in anaerobic planktonic culture in LB medium is ca. 0.03% final). The presence of 0.01%, 0.1%, 0.5% and 1% 1-propanol (final concentration) had no detectable growth impact on E. coli strain TG1. (TIF) [file pgen.1006800.s011.tif]

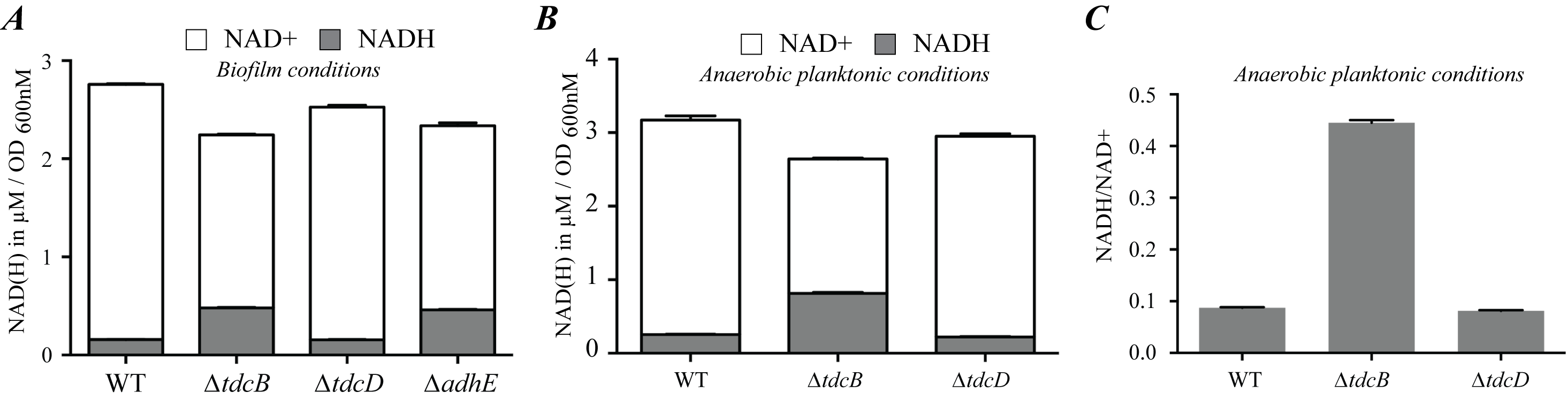

Supplement: S12 Fig — (A) NADH and NAD+ concentrations for E. coli wild-type (WT), ΔtdcB, ΔtdcD and ΔadhE mutant cultures grown under biofilm conditions in LB medium. (B) NADH and NAD+ concentrations for E. coli WT, ΔtdcB and ΔtdcD mutants cultures grown under anaerobic planktonic conditions in LB medium. In these conditions, the ΔadhE mutant exhibits a strong growth defect and cannot be meaningfully tested. (C) Impact of E. coli 1-propanol pathway on redox balance in LB medium under anaerobic planktonic conditions. Error bars represent the standard deviations from triplicate samples. (TIF) [file pgen.1006800.s012.tif]

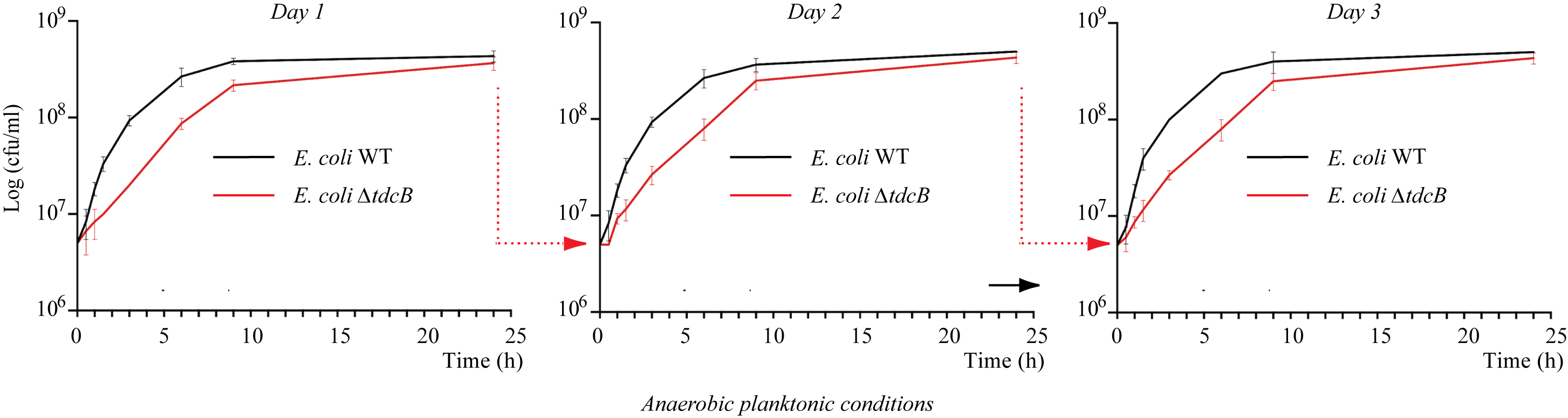

Supplement: S13 Fig — Comparison of E. coli ΔtdcB growth lag upon successive sub-cultivation show that E. coli ΔtdcB growth lag is consistently observed compared to wild type, indicating that ΔtdcB mutant recovery at 25H is not due accumulation of adaptive mutations. Successive subculture performed in triplicate in anaerobic planktonic conditions. (TIF) [file pgen.1006800.s013.tif]

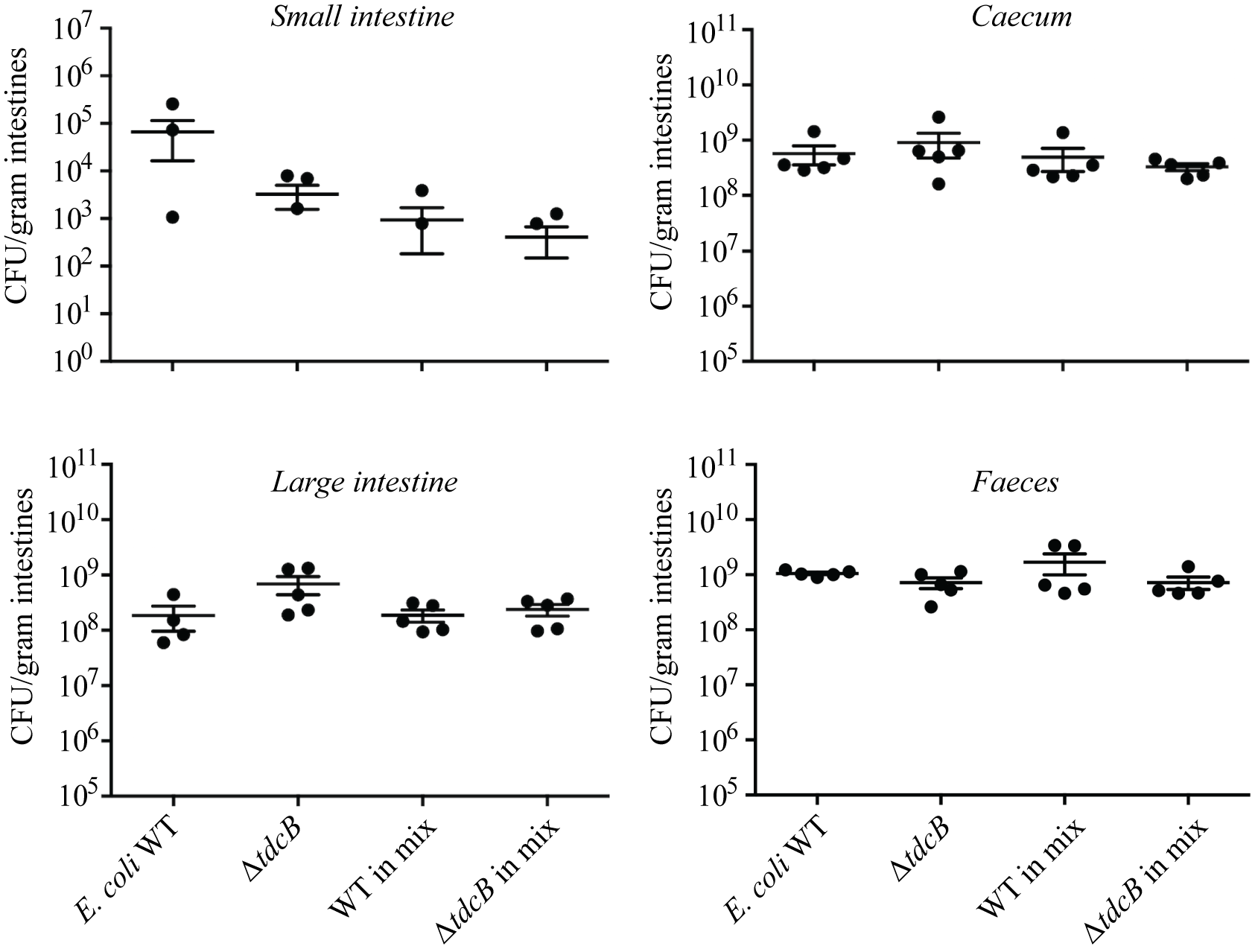

Supplement: S14 Fig — E. coli WT strain or tdcB mutant, alone or in mixed competition experiments performed in vivo show no significant decreased fitness of a tdcB mutant compared to E. coli WT. Colonization of small intestine by both E. coli strains is highly variable since 2 to 3 mice per conditions (10 mice in total) were not colonized in this section, so the corresponding values are not included on the graph. (TIF) [file pgen.1006800.s014.tif]
