# Supplementary material for: Biofilm microenvironment induces a widespread adaptive amino-acid fermentation pathway conferring strong fitness advantage in Escherichia coli
Source: PLoS Genet. 2017 May 19;13(5):e1006800. doi: 10.1371/journal.pgen.1006800 (PMC5459495; doi:10.1371/journal.pgen.1006800)
Supplement: S1 Table — (PDF) [file pgen.1006800.s015.pdf]

TABLE S1.

Complete list of *E. coli* K-12 mutants tested in this study and their impact on HS-SPME/GC-MS propanol signal.

**Mutants with no or strongly impaired[1] 1-propanol HS-SPME/GC-MS signal**
adhE, tdcB, crp, cyaA, fnr, tdcC, ***tdcE***
**Mutants with partial[2] 1-propanol HS-SPME/GC-MS signal**
rng, nadB
**Mutants with wild-type[3] 1-propanol HS-SPME/GC-MS signal**
eutG, fucO, yqhD, yiaY, glpQ, ackA, atoA, atoD, ilvI, ilvB, tynA, ilvA, leuA, mhpE, gldA, gcl, yfdU, poxB, tdh, ydjG, pta, tdcD, pflB, thrB, rcsB, pspF, phoP, slyA, evgA, lexA, rpoS, hns, arcA, soxR, cpxR, oxyR, aspC, luxS, relA, recA, fruR, ltaE, kbL, fis

In bold: mutants with severe biofilm formation defect preventing meaningful comparison with wild-type 1-propanol production in biofilm conditions.

[1] <30% of WT HS-SPME/GC-MS signal

[2] 30-80% of WT HS-SPME/GC-MS signal

[3] ≥85% of WT HS-SPME/GC-MS signal
