# Supplementary material for: Biofilm microenvironment induces a widespread adaptive amino-acid fermentation pathway conferring strong fitness advantage in Escherichia coli
Source: PLoS Genet. 2017 May 19;13(5):e1006800. doi: 10.1371/journal.pgen.1006800 (PMC5459495; doi:10.1371/journal.pgen.1006800)
Supplement: S2 Table — (PDF) [file pgen.1006800.s016.pdf]

Table S2.

List of strains used in this study

Strains and plasmids	Relevant characteristics	References[a]
**Strains**		
Escherichia coli		
TG1	Biofilm-forming *E. coli* K12; F'(*traD*36 *proAB* + *lacI*^{q} *lacZ*ΔM15 *supE hsd*Δ5 *thi* Δ(*lac-proAB*))	
MG1655F'	*E. coli* K12 F'*tet*	
55989	Enteroaggregative *E. coli*	(1)
O42	Enteroaggregative *E. coli*	(2)
536	Uropathogenic *E. coli*	(3)
CFT073	Uropathogenic *E. coli*	(4)
Other strains		
Salmonella Enterica	*S .enterica subsp. enterica*. human feces.	
Shigella flexneri	Serotype 2a. Isolated from human feces	(5)
Citrobacter rodentium 461	Isolated from hamster feces. CIP 104675	CRBIP
Pseudomonas aeruginosa	Strain *PAO1*	
Staphylococcus aureus	Strain HG001	
Enterobacter cloacae	CIP 108488	CRBIP
Aeromonas hydrophila	CIP 107274	CRBIP
Proteus mirabilis		CRBIP
Klebsiella pneumonia	Strain KP21	(6)

[a] Bacterial strains from our laboratory collection unless otherwise specified.
CRBIP**:** *Institut Pasteur, Centre de Resources Biologiques de l'Institut Pasteur.*

1. Da Re S, Valle J, Charbonnel N, Beloin C, Latour-Lambert P, Faure P, et al. Identification of commensal Escherichia coli genes involved in biofilm resistance to pathogen colonization. PLoS One. 2013;8(5):e61628. PubMed PMID: 23667443. Pubmed Central PMCID: 3646849.
2. Nataro JP, Deng Y, Cookson S, Cravioto A, Savarino SJ, Guers LD, et al. Heterogeneity of enteroaggregative Escherichia coli virulence demonstrated in volunteers. J Infect Dis. 1995 Feb;171(2):465-8. PubMed PMID: 7844392. Epub 1995/02/01. eng.
3. Berger H, Hacker J, Juarez A, Hughes C, Goebel W. Cloning of the chromosomal determinants encoding hemolysin production and mannose-resistant hemagglutination in Escherichia coli. J Bacteriol. 1982 Dec;152(3):1241-7. PubMed PMID: 6754701. Pubmed Central PMCID: 221631. Epub 1982/12/01. eng.
4. Mobley HL, Green DM, Trifillis AL, Johnson DE, Chippendale GR, Lockatell CV, et al. Pyelonephritogenic Escherichia coli and killing of cultured human renal proximal tubular epithelial cells: role of hemolysin in some strains. Infect Immun. 1990 May;58(5):1281-9. PubMed PMID: 2182540. Pubmed Central PMCID: 258621. Epub 1990/05/01. eng.
5. Jin Q, Yuan Z, Xu J, Wang Y, Shen Y, Lu W, et al. Genome sequence of Shigella flexneri 2a: insights into pathogenicity through comparison with genomes of Escherichia coli K12 and O157. Nucleic Acids Res. 2002 Oct 15;30(20):4432-41. PubMed PMID: 12384590. Pubmed Central PMCID: 137130.
6. Favre-Bonte S, Joly B, Forestier C. Consequences of reduction of Klebsiella pneumoniae capsule expression on interactions of this bacterium with epithelial cells. Infect Immun. 1999 Feb;67(2):554-61. PubMed PMID: 9916058. Pubmed Central PMCID: 96354.
