# Supplementary material for: Biofilm microenvironment induces a widespread adaptive amino-acid fermentation pathway conferring strong fitness advantage in Escherichia coli
Source: PLoS Genet. 2017 May 19;13(5):e1006800. doi: 10.1371/journal.pgen.1006800 (PMC5459495; doi:10.1371/journal.pgen.1006800)
Supplement: S3 Table — (PDF) [file pgen.1006800.s017.pdf]

TABLE S3.

Complete list of oligonucleotide primers used to perform qRT-PCR experiments in this study.

Target gene	Primer name	Primer sequence - from 5' to 3'
16S (*rssH*)	TM1[11]	atgaccagccacactggaac
	TM2[11]	cttcctccccgctgaaagta
acka	acka_F	ggtggttccgtttctgctat
	acka_R	tcttcaacatagcggcagtc
adhE	adhe_F	ggcgtaatctgtgcttctga
	adhe_R	cagctctttaccctgcaaca
hcaT	hcaT_F[12]	gctgctcggctttctcatcc
	hcaT_R[12]	ccaaccacgcagaccaacc
ihfB	ihfb_F[12]	gcggtttcggcagtttct
	ihfb_R[12]	cgcagttctttaccaggttt
ilva	ilva_3F	cggaaggtgccgaatattta
	ilva_3R	aagacgcgacgacagttttt
opgG	opgg_F	tgcacgactctaacggtctc
	opgg_R	agatcttcaaagcgggagaa
pflb	pflb_F	acgcagtacgtttcaactcg
	pflb_R	tcgagtctgttttggcagtc
pta	pta_3F	gcgcgcatttctaaactgt
	pta_3R	tgatcgtcaaccggaactt
tdcB	tdcB_F	ggcggtttaattgctggtat
	tdcB_R	ggttatttctccggagtgga
tdcD	tdcD_F	acacgcagcttcattacgtc
	tdcD_R	cgctcaccacaggagttaga
tdcE	tdcE_2F	cgcattatcggtgactatcg
	tdcE_2R	tttcctgaatctgcaacagc
